# Genome assembly and venom gene mapping in the medically important moth *Lonomia casanarensis* (Saturniidae: Hemileucinae)

**DOI:** 10.1093/g3journal/jkag113

**Published:** 2026-05-13

**Authors:** Diler Haji, Samridhi Chaturvedi, Oanh H Nguyen, Noravit Chumchim, Diana M Toro-Vargas, Juana Díaz-Díaz, Lina V Lozano-Morales, Gustavo A Bravo, Camila González, Noah K Whiteman

**Affiliations:** Department of Integrative Biology, University of California, Berkeley, CA 94720, United States; Department of Ecology and Evolutionary Biology, Tulane University, New Orleans, LA 70118, United States; DNA Technologies and Expression Analysis Core Laboratory, Genome Center, University of California, Davis, CA 95616, United States; DNA Technologies and Expression Analysis Core Laboratory, Genome Center, University of California, Davis, CA 95616, United States; Centro de Investigaciones en Microbiología y Parasitología Tropical-CIMPAT, Departamento de Ciencias Biológicas, Facultad de Ciencias, Universidad de Los Andes, Bogotá 111711, Colombia; Centro de Investigaciones en Microbiología y Parasitología Tropical-CIMPAT, Departamento de Ciencias Biológicas, Facultad de Ciencias, Universidad de Los Andes, Bogotá 111711, Colombia; Centro de Investigaciones en Microbiología y Parasitología Tropical-CIMPAT, Departamento de Ciencias Biológicas, Facultad de Ciencias, Universidad de Los Andes, Bogotá 111711, Colombia; Department of Organismic and Evolutionary Biology & Museum of Comparative Zoology, Harvard University, Cambridge, MA 02138, United States; Centro de Colecciones y Gestión de Especies, Instituto Alexander von Humboldt, Villa de Leyva 154001, Colombia; Centro de Investigaciones en Microbiología y Parasitología Tropical-CIMPAT, Departamento de Ciencias Biológicas, Facultad de Ciencias, Universidad de Los Andes, Bogotá 111711, Colombia; Department of Integrative Biology, University of California, Berkeley, CA 94720, United States; Department of Molecular & Cell Biology, University of California, Berkeley, CA 94720, United States

**Keywords:** *Lonomia casanarensis*, *Lonomia obliqua*, Saturniidae, envenoming, stinging, venom, hemorrhagic syndrome, Colombia, caterpillar, genome assembly

## Abstract

Caterpillars of *Lonomia* moths (Saturniidae) are among the most medically significant lepidopterans worldwide. Their envenomation causes a severe hemorrhagic syndrome that can be fatal. Although antivenom therapy exists it is only produced using *Lonomia obliqua* venom extract. More than 60 additional species of *Lonomia* are distributed across Central and South America, many of which pose an uncharacterized risk to human health. To enable comparative venom studies and genome-guided discovery of toxin genes, we present the first genome assembly of *Lonomia casanarensis*, a species responsible for most envenomation cases in Colombia. The assembly, generated using PacBio Revio long-read sequencing, spans 483.4 Mb with a scaffold N50 of 7.7 Mb, N90 of 2.4 Mb, and >99% BUSCO completeness. It comprises 154 contigs organized into 118 scaffolds and a complete mitochondrial genome. Phylogenomic analyses based on 1,190 single-copy orthologs place *L. casanarensis* within a clade of scoli-bearing saturniids. We provide a genomic map of the 21 venom genes previously identified from *L. obliqua* transcriptomic and proteomic data. This high-quality genome provides a foundation for the development of improved antivenoms and facilitates exploration of novel bioactive compounds from *Lonomia* venoms.

## Teaser

Haji *et al*. present the first high-quality genome assembly of a *Lonomia* species (*L. casanarensis*), which contains venomous saturniid caterpillars responsible for hemorrhagic envenomation cases and is of major biomedical importance. Using PacBio Revio long-read sequencing, the authors generated a 483.4 Mb assembly with exceptional contiguity (scaffold N50 = 7.7 Mb and N90 = 2.4 Mb) and completeness (>99% BUSCO). The authors further identified and mapped venom gene homologs previously characterized in the well-studied L. obliqua, integrating these results with phylogenomic analyses across Saturniidae. Together, these resources illuminate the genetic architecture of venom evolution in this medically significant genus.

## Introduction

Larvae of Neotropical moths in the genus *Lonomia* Walker, 1855 (Saturniidae) possess cuticular spines called scoli, which are modified setae capable of injecting venom (Spadacci-Morena et al. 2016). These caterpillars pose a significant public health risk because their venom disrupts coagulation in humans who accidentally come into contact with the spines, typically by brushing against aggregated larvae resting on tree trunks. *Lonomia* caterpillars are nocturnal folivores that form dense, gregarious clusters during the day, a behavior that increases the risk of human contact (González et al. 2023). Envenomation by *Lonomia* spp. can cause a hemorrhagic syndrome characterized by the simultaneous activation and inhibition of clotting pathways, leading to systemic bleeding, renal failure, neurological damage, and occasionally death (Gamborgi et al. 2006; Pinto et al. 2010). Thousands of human envenomation cases are reported annually across the Neotropics. For example, Brazil alone recorded 6,636 cases between 2007 and 2018 (Favalesso et al. 2021). Although *L*onomia *achelous* Cramer, 1777 and *Lonomia obliqua* Walker, 1855 are the most studied species, at least 60 *Lonomia* species are now recognized across Central and South America, including several in Colombia (González et al. 2023).

An antivenom developed by the Butantan Institute (Brazil) for *L. obliqua* venom has been used successfully to treat envenomation by that species and several congeners (Da Silva et al. 1996; Sano-Martins et al. 2018). However, there are likely considerable differences in the venom components between species, suggesting that novel antivenoms and other strategies for treatment are needed. For example, the effects of venom on the coagulation pathway differ between species: *L. obliqua* venom has mostly pro-coagulant activity, whereas *L. achelous* venom has both pro- and anti-coagulant activity, with a particularly significant impact on fibrinolysis (Carrijo-Carvalho and Chudzinski-Tavassi 2007). Variation in venom composition may also affect the effectiveness of treatment, as the antifibrinolytic drug ε-aminocaproic acid, which has been successfully used to treat *L. achelous* envenoming (Arocha-Piñango et al. 1992), has been shown to increase mortality rates in rats envenomed with *L. obliqua* venom (Gonçalves et al. 2007). Sano-Martins et al. (2018) also demonstrated that the recuperation rate of blood fibrinogen levels was slower in rats envenomed with *Lonomia casanarensis* venom than in those envenomed with *L. obliqua* when treated with Butantan Institute antivenom. Finally, regarding variation in venom composition within the genus, Coppens (2016) suggests that the secretion of *Lonomia electra* Druce, 1886 caterpillars might not be poisonous at all, causing only skin irritation. The lack of overlap between *Lonomia* envenoming cases and the distribution of some species in the *L. electra* phylogenetic group (González et al. 2023) also supports this hypothesis.

In parallel, *Lonomia* venom presents opportunities for pharmacological discovery. Several venom proteins exhibit thrombolytic or anticoagulant activities relevant to cardiovascular disease treatment. For example, Lonomin V, a fibrinolytic protein from *L. achelou*s venom, has been proposed as a useful tool for developing antithrombotic agents (Guerrero et al. 2001, 2011). In addition, other compounds derived from *Lonomia* spp. have been studied for their potential use in cell culture, and antiviral and antibiotic treatments among others (Riva and Amarillo-S 2023). Therefore, genomic resources of the genus are useful for addressing and increasing public health threat of envenomation and guiding future efforts of drug development.

In Colombia, *L. casanarensis* Brechlin, 2017 has been confirmed through DNA barcoding as the species responsible for envenomation incidents in Casanare Department, including the country's first recorded fatal case (Pineda et al. 2001; González et al. 2023). This species has also been found in Arauca and Meta, departments that, together with Casanare, form part of the Colombian Llanos region. In addition, experiments with this species have demonstrated that it induces hemostatic disturbances in rats, further underscoring its medical importance (Da Silva et al. 1996; Sano-Martins et al. 2018).

Here, we present the first nuclear and mitochondrial genome assemblies for *L. casanarensis*, a deadly species from Colombia. Using comparative genomic and phylogenomic analyses, we placed this lineage among other Saturniidae moths and identified putative venom gene homologs by mapping *L. obliqua* venom transcripts (Veiga et al. 2005) to the assembly. This genome provides a foundational resource for public health applications and for the exploration of novel therapeutic compounds derived from *Lonomia* spp. venoms.

## Materials and methods

### Sample collection, DNA extraction, and sequencing

**Fig. 1.**
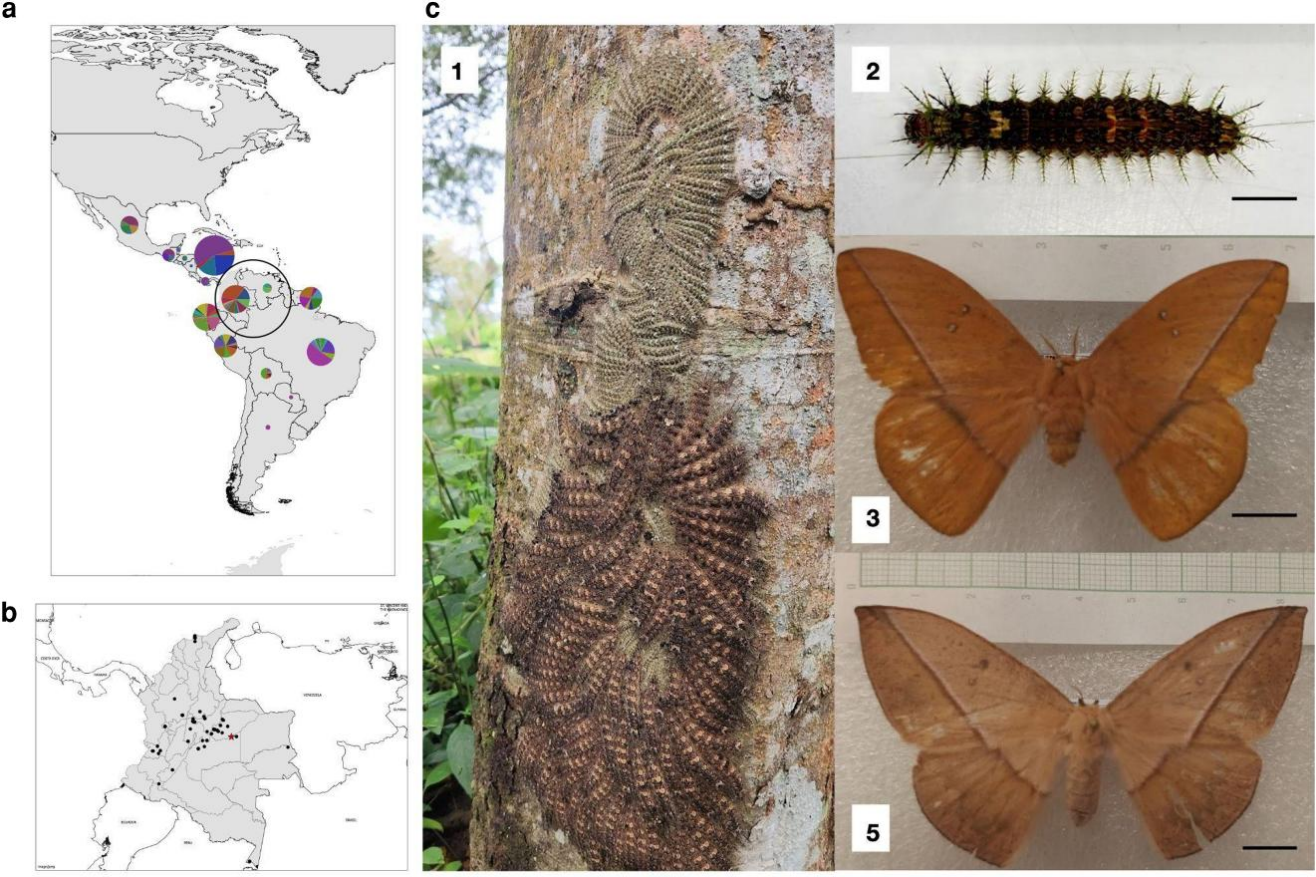
Species richness and distribution maps of the *Lonomia* genus. a) The map shows the species richness of *Lonomia* across its entire range on the American continent. Black circle indicates the area of Colombia. b) A zoomed-in map of the distribution of *Lonomia* spp. in Colombia and the localities where it was collected. The star marks the location where the sample for sequencing the current genome sequence was obtained. c) Life cycle of *L. casanarensis*. (C1) Caterpillar colonies in the fourth instar (top) and sixth instar (bottom) in resting position on a tree trunk. (C2) Caterpillar in the sixth/final instar. (C3) Adult male. (C4) Adult female. Scale bar = 1 cm. Maps were done using ArcGis (Licensed to Uniandes). Country border shape file available at: https://gadm.org/data.html.

A colony of 14 *Lonomia* sp. caterpillars were collected on 2020 November 16, in the Department of Meta (San Pedro de Arimena, Puerto Gaitán municipality), Colombia (Fig. 1). The individuals were in the fourth and fifth larval instars and were transported alive to the Centro de Investigaciones en Microbiología y Parasitología Tropical, Universidad de Los Andes, for species identification and further processing.

Caterpillars were identified, following the method previously described by Toro-Vargas et al. (2023), which uses instar-specific morphological traits for larval staging and preliminary species assignment. Total genomic DNA was extracted from 2 prolegs using the Quick-DNA Tissue/Insect MiniPrep Kit (Zymo Research, USA). A 658 bp fragment of the *cytochrome c oxidase subunit I* (*COI*) gene was amplified with the standard lepidopteran barcode primers LepF1/LepR1. The amplification product (sample ID: CGR_Lon178) was Sanger sequenced, and the resulting sequence was compared using BLAST against reference records in the Barcode of Life Data Systems (BOLD). Species identity was confirmed as *L. casanarensis* with >98% sequence similarity to reference barcodes (González et al. 2023).

For genome sequencing, high-molecular-weight (HMW) genomic DNA (gDNA) was isolated from a single frozen *L. casanarensis* caterpillar (a small section of the abdomen including prolegs and spines was used as tissue) sent to and processed at the University of California, Berkeley, using the Qiagen MagAttract HMW DNA Kit (Qiagen, Germany). Specimens were collected under Collection Permit Resolution 1177 of 2014 October 9 and sample shipment was authorized under the 02498-export permit issued by the Colombian Environmental Agency (ANLA) to Universidad de Los Andes. The quality of the extracted gDNA was assessed using fluorometric quantification on a Qubit 3.0 fluorometer (Thermo Fisher Scientific, Inc.). The DNA concentration was estimated at 21.6 ng/µL in a total volume of 15 µL, corresponding to approximately a mass of 324 ng of DNA. The integrity of the gDNA was evaluated using an Agilent Bioanalyzer, which revealed HMW fragments ranging from 10 to 100 kb, suitable for long-read genomic sequencing. The gDNA isolate was then outsourced to the UC Davis Sequencing Core for library preparation and PacBio whole-genome sequencing.

### HiFi SPK3 library preparation and PacBio sequencing

High-fidelity (HiFi) SMRTbell libraries were constructed using the SMRTbell Prep Kit 3.0 (Pacific Biosciences, Menlo Park, CA, USA; Cat. #102-182-700) following the manufacturer's protocol. HMW genomic DNA was sheared to a target fragment size of 15 to 20 kb using a Megaruptor 3 system (Diagenode, Belgium; Cat. #B06010003). The sheared DNA was concentrated with 1× SMRTbell cleanup beads provided in the kit, followed by DNA repair and A-tailing at 37 °C for 60 min and 65 °C for 5 min. Overhang adapters were ligated at 20 °C for 30 min, and the resulting SMRTbell libraries were purified using 1× SMRTbell cleanup beads, followed by nuclease treatment at 37 °C for 15 min to remove damaged or unligated DNA. Libraries were size-selected with 3.1× volumes of 35% (v/v) diluted AMPure PB beads (Pacific Biosciences, Cat. #100-265-900) to progressively remove fragments <5 kb. The final libraries were sequenced on the PacBio Revio platform using one 25 M SMRT Cell, with 30-h movie collections per cell.

### Genomic data quality assessment

K-mer based analyses were performed to estimate genome characteristics and assess assembly quality. K-mer counts were generated from raw PacBio HiFi reads using *meryl* (https://github.com/marbl/meryl), and these counts were analyzed with GenomeScope2.0 (Ranallo-Benavidez et al. 2020) to estimate genome size, heterozygosity, and repeat content. To evaluate contiguity and structural completeness of both the sequencing data and the assembled genome (see below), we used QUAST v5.2.0 (Gurevich et al. 2013). Assembly completeness and gene-space representation were assessed with BUSCO v5.7.1 (Simão et al. 2015) using the Lepidoptera_odb10 dataset, which includes 1,367 conserved single-copy orthologs. Finally, base-level accuracy (QV) and k-mer completeness were evaluated using *merqury* (Rhie et al. 2020) with the *meryl*-derived k-mer database.

### Genome assembly pipeline and quality assessment

#### Nuclear genome assembly

The *L. casanarensis* genome sequence was assembled using a custom PacBio HiFi assembly pipeline, incorporating optimized tools and parameters for high-contiguity genome reconstruction. Residual adapter sequences were first removed from the PacBio HiFi reads using HiFiAdapterFilt (Sim et al. 2022). A haplotype-resolved diploid genome assembly was then generated with *Hifiasm* v0.19.9-r616 (Cheng et al. 2022) in default mode. *Hifiasm* generated 2 haplotype assemblies, each representing a different haplotype in the diploid genome. The assembler phases reads de novo by exploiting heterozygous variants in the HiFi data, enabling haplotype resolution without parental information. Assembly quality was initially evaluated using BUSCO v5.7.1 (Simão et al. 2015) with the Lepidoptera_odb10 ortholog dataset and QUAST v5.2.0 (Gurevich et al. 2013) for contiguity and summary metrics. To benchmark our results, we also applied *compleasm* v0.2.5 (Huang and Li 2023) to publicly available Saturniidae genomes deposited in GenBank, using selected Sphingidae species as outgroups (see phylogenomic analysis below).

Preliminary assessments revealed duplicated regions and unresolved pseudo-haplotypes in the draft assemblies. To mitigate these issues, we used *purge_dups* v0.0.3, executing *pd_config.py* followed by *run_purge_dups.py* with default parameters. Post-processing with BUSCO and QUAST confirmed a substantial reduction in duplicated content. The resulting deduplicated assemblies were designated as the primary assemblies and further assessed for completeness and heterozygosity using the BlobToolKit Framework v4.4.0 (Challis et al. 2020), Scaffolding was performed with SALSA (Ghurye et al. 2017), using alignment files generated by Minimap2 (Li 2018) that mapped the deduplicated contigs against the raw HiFi reads. The final scaffolded assembly was re-evaluated with BlobToolKit, BUSCO, and *compleasm* (v0.2.5) using the Lepidoptera_odb10 dataset to confirm structural integrity and gene completeness. Comparative *compleasm* analyses with other Saturniidae and Sphingidae genomes further validated assembly quality and completeness.

#### Nuclear genome annotation

We annotated protein-coding genes in the *L. casanarensis* PacBio HiFi genome assembly using the BRAKER3 pipeline (v3.0.8) in protein-supported mode. Prior to annotation, we identified repetitive elements de novo using RepeatModeler (v2.0.7) and masked them using RepeatMasker (v4.2.3) to generate a soft-masked genome assembly. We compiled protein evidence from multiple Lepidoptera species, including *Bombyx mori*, *Danaus plexippus*, *Manduca sexta*, and *Automeris io*, and clustered these sequences at 95% sequence identity using CD-HIT (v4.8.1) to reduce redundancy in the input protein dataset. We then used BRAKER3, which integrates GeneMark-EP+ and AUGUSTUS, to predict gene models using protein homology hints generated by ProtHint. The resulting gene models were exported in GFF3 and GTF formats along with predicted coding sequences and protein translations. To reduce redundancy arising from alternative transcript isoforms, we retained only the longest isoform per gene using AGAT (v1.6.1). Structural annotation statistics were calculated using AGAT, which was used to summarize gene, transcript, exon, and CDS features from the BRAKER output, including total gene and transcript counts, exon and CDS counts, and distributions of gene, exon, intron, and coding sequence lengths. We assessed annotation completeness using BUSCO (v5) with the lepidoptera_odb10 dataset. To further evaluate redundancy in the predicted protein set, we clustered BRAKER-derived protein sequences at high sequence identity using CD-HIT and reran BUSCO on the resulting non-redundant protein set. This diagnostic analysis was used to distinguish redundancy in predicted gene models from true biological duplication. Functional annotation of predicted proteins was performed using InterProScan (v5.77), integrating multiple protein signature databases including Pfam, SUPERFAMILY, and Gene3D to assign conserved domains, InterPro identifiers, and Gene Ontology (GO) terms. InterProScan results were summarized using custom scripts to quantify the number and proportion of proteins with functional annotations, the number of unique InterPro and Pfam domains, and the proportion of proteins associated with GO terms. Orthology-based functional annotation was additionally performed using eggNOG-mapper (v2) in DIAMOND mode against the eggNOG v5 database to assign functional descriptions, GO terms, and KEGG pathway annotations. Together, these approaches provided a comprehensive structural and functional annotation of the *L. casanarensis* genome while enabling evaluation of annotation redundancy and completeness.

#### Mitochondrial genome assembly

We identified scaffold 117 of the *L. casanarensis* haplotype 1 assembly as the mitochondrial genome based on BLAST searches using reference mitochondrial genomes from other Lepidopteran species. To confirm and refine this identification, we independently assembled the mitochondrial genome using MitoHiFi (Uliano-Silva et al. 2023). A suitable mitochondrial reference sequence for annotation and assembly was selected with the findMitoreference.py script provided in the MitoHiFi package. We specified *Hylesia metabus* as the reference species because both our genome-wide nuclear phylogeny and CO1 mitochondrial phylogeny (see below) placed *H. metabus* as the sister lineage to *L. casanarensis* among available Saturniidae genomes in GenBank. Among the *H. metabus* mitochondrial genomes available, we selected a reference sequence ≥14,000 bp in length to ensure near-complete gene representation. All PacBio HiFi reads were then provided as input to the mitohifi.py script for mitochondrial genome assembly and annotation, with automated gene prediction performed using MITOS (Bernt et al. 2013) (Supplementary Figs. 2 and 3). The mitochondrial genome assembly procedure is specified in the Snakemake pipeline (*mito.smk*).

#### Phylogenomics and venom genes

Single-copy orthologous genes were extracted from the *L. casanarensis* genome assembly and from publicly available genome assemblies representing other Saturniidae species and outgroup Sphingidae. Orthologs were identified using *compleasm* v0.2.6 (v0.2.6) (Huang and Li 2023) with the lineage specified as Lepidoptera. We retained genes that were present in at least 15 genome assemblies for downstream phylogenetic analysis to maximize information content across our alignment. Individual protein (amino-acid) sequences produced by the *compleasm* pipeline were aligned using MAFFT V7.490 (Katoh and Standley 2013) with the algorithm selections set to automatic. We inferred maximum likelihood gene trees for each alignment using IQ-TREE v1.6.12 (Minh et al. 2020). Best-fit substitution models were determined independently for each locus using ModelFinder (Kalyaansmoorthy et al. 2017) based on the Bayesian Information Criterion. The specific model selected for each gene is provided in Supplementary Table 2. The resulting gene trees were then used as input to ASTRAL v5.7.8 (Zhang et al. 2018) to reconstruct a coalescence-based species tree. The phylogenetic inference procedure is specified in the Snakemake pipeline (*compleasm.smk* and *tree.smk*).

Separately, we reconstructed a mitochondrial phylogeny using *CO1* sequences from the BOLD. We minimized taxonomic redundancy by collapsing subspecies and selecting a single representative sequence per species based on maximum sequence length and minimal ambiguous base content. The dataset was filtered to retain sequences between 600 and 900 bp and aligned in MAFFT V7.490. We inferred a maximum likelihood tree using this *CO1* nucleotide alignment in IQ-TREE with default settings and nodal support was evaluated using 100 bootstrap replicates.

We performed pairwise genome alignments using minimap2 v2.24 (Li 2021) with assembly-to-assembly alignment mode. We used the high sequence divergence preset (asm20) for alignments between species and a low sequence divergence preset (asm5) for alignments between the 2 haplotypes of the *L. casanarensis* assembly. We initially generated the alignment in SAM format and then used *paftools.js* script to convert the alignments to PAF format for downstream analysis. We generated another set of mapping files representing the genomic positions of previously identified venom transcript sequences (Veiga et al. 2005) using the *splice* preset in *minimap2* to generate splice-aware alignments. Using a custom *R* script, we generated dotplots to visualize the mapping between genomes and haplotypes overlaid with the genomic coordinates of venom transcript sequences. The genome alignment procedure is specified in the Snakemake pipeline (*minimap.smk*) and the alignment visualization is specified in the custom script *synteny.R*.

Similarly, we used *minimap2* (v2.24) with the splice-aware mapping mode to align previously published complimentary DNA (cDNA) sequences from *L. obliqua* (Veiga et al. 2005) to the *L. casanarensis* genome assembly. This enabled identification of exon-intron structures, which were then used to annotate downstream exon alignments. The *L. casanarensis* exon sequences were concatenated into a single coding sequence and aligned to their *L. obliqua* cDNA homolog using MAFFT (v7.490). Reading frames were manually verified for each alignment and blastp searches were performed against the default clusteredNR database. Protein sequences corresponding to the top 10 hits with the lowest *E*-value were subsequently aligned to the translated *L. casanarensis* and *L. obliqua* sequences. Representative alignments are shown in Fig. 3, with a complete catalogue provided in Supplementary Figs. 4 to 37. Alignment visualizations were generated using ESPript 3.0 (Gouet et al. 2003). The mapping procedure is specified in the Snakemake pipeline (*minimap.smk*).

## Results and discussion

### Genome assembly and annotation

**Fig. 2.**
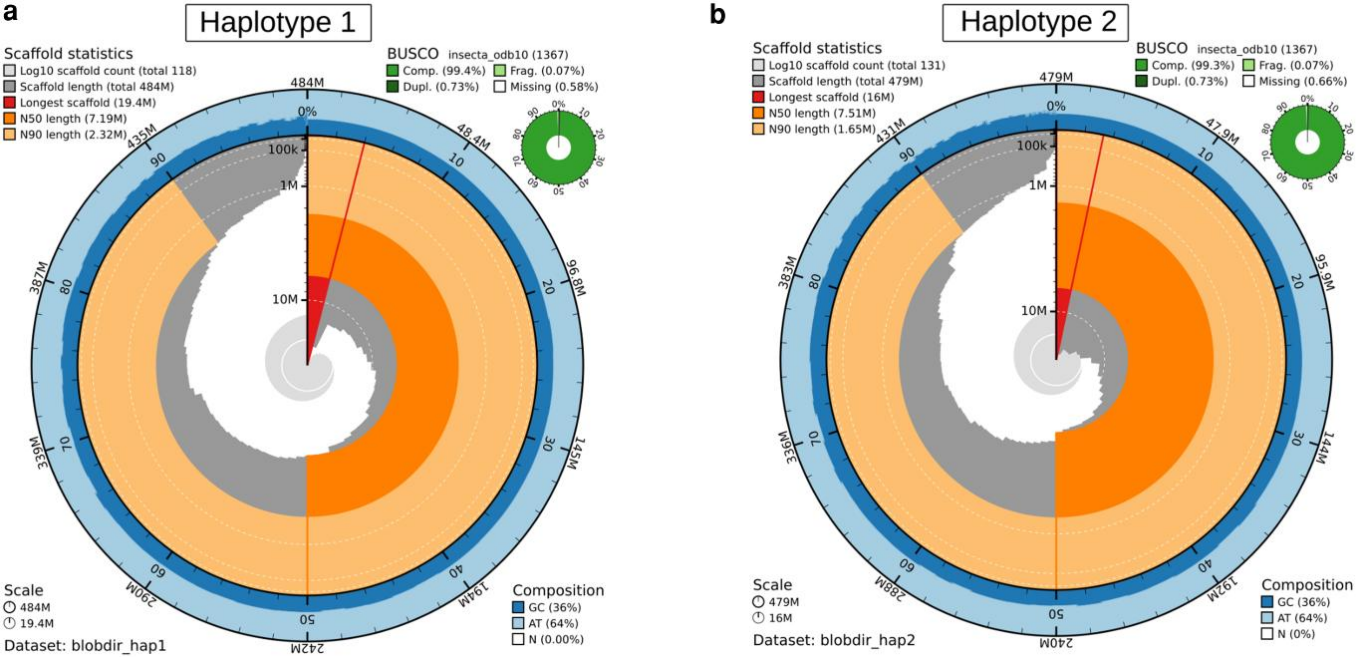
BlobToolKit Snail plot showing a graphical representation of the quality metrics presented in Supplementary Table 1 for the *L. casanarensis* genome assembly for (a) haplotype 1, and (b) haplotype 2. In both plots, the plot circle indicates the full size of the assembly. From the inside out, the central plot displays length-related metrics. The red line shows the length of the longest scaffold, while all other scaffolds are arranged in decreasing size in a clockwise direction around the plot, starting from the outside of the central circle and drawn in gray. Dark and light orange arcs depict the scaffold N50 and N90 values, respectively. The central light gray spiral illustrates the cumulative scaffold count, with a white line marking each order of magnitude. White areas indicate the proportion of Ns in the assembly, and the surrounding dark and light blue regions represent the mean, maximum, and minimum GC versus AT content at 0.1% intervals (Challis et al. 2020). BUSCO completeness scores are indicated in the top right of each figure panel. The score is 99.4% for haplotype 1 and 99.3% for haplotype 2.

PacBio Revio sequencing generated 3,889,484 HiFi read pairs, yielding ∼94× coverage based on the GenomeScope2.0 genome size estimate of 422.89 Mb. From the HiFi reads, we estimated a 0.18% sequencing error rate and a 3.37% nucleotide heterozygosity rate. The k-mer spectrum exhibited a clear bimodal distribution with peaks near 94× and 190× coverage (Fig. 2a), corresponding to heterozygous and homozygous k-mers, respectively. This pattern indicates a highly heterozygous diploid genome.

The final nuclear genome assembly consisted of 2 haplotypes (Hap1 and Hap2), each similar in size to the GenomeScope2.0 estimate and consistent with QUAST summary statistics. Haplotype 1 comprised 118 scaffolds and 154 contigs, spanning 483.4 Mb, with a contig N50 of 6 Mb, scaffold N50 of 7.19 Mb, longest contig 19.37 Mb, and largest scaffold 19.4 Mb. Haplotype 2 comprised 131 scaffolds and 131 contigs, spanning 479.3 Mb, with a contig N50 of 7 Mb, scaffold N50 of 7.5 Mb, longest contig 15.98 Mb, and largest scaffold 15 Mb. Comprehensive assembly statistics are summarized in Supplementary Table 1, with graphical representations shown for haplotype 1 (Fig. 2a) and haplotype 2 (Fig. 2b).

Assembly completeness was high, with >99% BUSCOs detected and only 0.7% duplicated complete genes, confirming the overall quality and low redundancy of both haplotypes (Fig. 2). The assembly metrics of *L. casanarensis* are broadly comparable to those of its closest available genome, *Automeris io* (Skojec et al. 2024), which has a genome size of 490 Mb, 37% GC content, and 98.4% BUSCO completeness, albeit with a higher N50 of 15.78 Mb. In contrast, the genome of *H. metabus* (Perrier et al. 2025), another medically important South American saturniid, is nearly 3 times larger due to extensive transposable-element accumulation.

We annotated the haplotype 1 genome assembly and summarized the major structural and functional features of the genome in Supplementary Table 3. We predicted a total of 33,191 protein-coding genes and 37,088 transcripts using BRAKER3. Gene models had an average of 5.82 exons per transcript, with mean gene, exon, intron, and coding sequence lengths of 7,127.1, 227.9, 1,279.4, and 1,327.1 bp, respectively. Structural annotation summaries indicated that 80.86% of transcripts were multi-exonic, while 19.14% were single-exon transcripts. BUSCO analysis using the Lepidoptera_odb10 dataset indicated high completeness of the annotation, with 97.7% complete BUSCOs (93.5% single-copy, 4.2% duplicated), 0.7% fragmented, and 1.6% missing. Initial BUSCO analysis of the unfiltered annotation showed elevated duplication, which was substantially reduced after clustering predicted proteins at high sequence identity, indicating that the initial duplication signal was driven primarily by redundant highly similar gene models rather than true biological duplication. Functional annotation using InterProScan identified conserved domains in 32,013 of 37,088 predicted proteins (86.32%), yielding 12,620 unique InterPro entries and 5,921 unique Pfam domains. Orthology-based annotation using eggNOG-mapper assigned functional descriptions to 30,815 proteins (83.09%) and identified 18,015 proteins associated with KEGG pathways. The mitochondrial genome assembled into a single, circularized sequence of 15,434 bp with a GC content of 20.8%, containing 12 protein-coding genes, 23 tRNA genes, and 2 rRNA genes.

### Phylogenomics and venom gene mapping

**Fig. 3.**
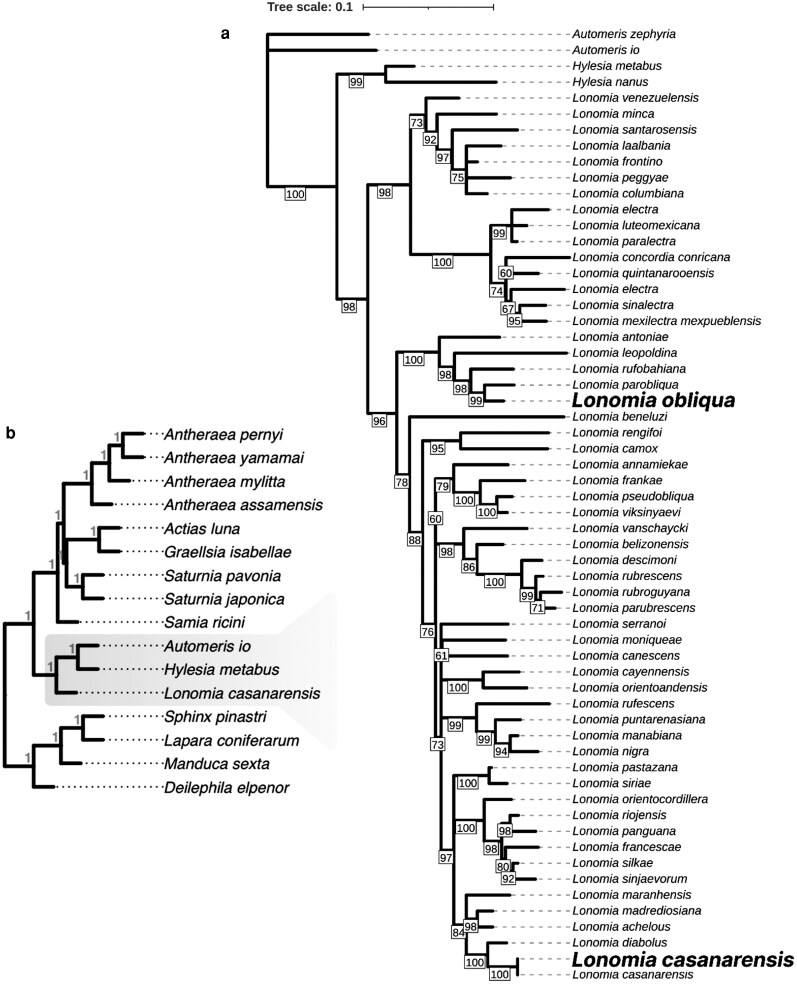
a) ASTRAL species tree inferred from maximum-likelihood amino acid phylogenies of 1,190 single-copy orthologs. b) Maximum likelihood mitochondrial tree (*COI*) of *Lonomia* species with outgroups. Node labels represent bootstrap support values (100 iterations).

Using 1,114 single-copy orthologs shared across *L. casanarensis* and related Saturniidae plus sphingid outgroups, we recovered a well-supported ASTRAL species tree that placed *L. casanarensis* as sister to a clade comprising *Automeris io* and *Hylesia metabus*. The placement of *A. io* differed between the nuclear and mitochondrial trees, suggesting mito-nuclear discordance. In the nuclear tree, *A. io* and *H. metabus* form a clade that is sister to *Lonomia*. In the mitochondrial tree, *A. io* is sister to clade formed by *H. metabus* and a monophyletic *Lonomia* lineage. In both cases, *H. metabus* is sister to *Lonomia* (Fig. 3). The phylogenomic context establishes a framework for exploring how venom repertoires have diversified within the genus *Lonomia* and across the Saturniidae. Phylogenetic analyses using ultraconserved elements across 338 species representing all described saturniid subfamilies, tribes, and genera have produced topologies similar to those represented in the nuclear species tree provided in this study (Rougerie et al. 2022). Traditionally, *Periga* has been considered the sister genus to *Lonomia*, however we did not include *Periga* in this study as there is no representative genome sequence available for species in this genus to date.

Mapping of *L. obliqua* venom cDNAs (Veiga et al. 2005) to the *L. casanarensis* genome recovered 19 genes spanning the major functional categories implicated in *Lonomia* envenomation, including proteases, protease inhibitors, lectins, lipocalins, and antimicrobial peptides (AMPs) (Table 1, see Supplementary Figs. 4 to 37 for a complete catalogue of detailed alignments). The scattering across many scaffolds suggests that venoms in *Lonomia* are encoded by a distributed repertoire rather than a discrete genomic cluster. Together, these results reveal a combination of conserved and divergent components of venom architecture relative to *L. obliqua*. Two cysteine protease homologs were identified which include a cathepsin B-like protease (LOqua-CysPep2, scaffold 7: 2.99 to 3.04 Mb) and a cathepsin L-like protease (LOqua-CysPep1, scaffold 30: 0.24 Mb). Their activity parallels the fibrinolytic and prohemorrhagic functions documented in *L. obliqua* venom (Pinto et al. 2010). A cystatin inhibitor (LOqua-Cyst1, scaffold 32: 2.84 to 2.84 Mb) was also detected, likely acting as an endogenous regulator to prevent premature proteolysis within the gland. Multiple trypsin- and chymotrypsin-like serine proteases were recovered, including LOqua-SP4 (scaffold 5: 2.41 to 2.42 Mb), LOqua-SP7 (scaffold 34: 3.81 Mb), and LOqua-SP3 (scaffold 9: 10.06 to 10.07 Mb), as well as a masquerade-like protease (LOqua-PPOAF1, scaffold 87: 0.81 Mb). These enzymes belong to the PPO-activating factor family and may drive fibrinolytic cascades involving the hemorrhagic effect (Guerrero et al. 2001). Three serpin genes (Serp1, Serp3, and Serp6) were identified on scaffolds 8, 12, and 15, respectively. In insects, serpins maintain hemolymph homeostasis by damping melanization and clotting cascades. In *Lonomia*, they may similarly regulate venom protease activity thereby preventing autolysis or fine-tuning host responses post-envenomation. Their distribution across distinct scaffolds suggests independent duplication events rather than tandem expansion. Four lectin-like sequences were mapped, including 2 lipopolysaccharide-binding proteins (LOqua-Lect1 and LOqua-Lect3, both on scaffold 7), an uncharacterized lectin-like gene (LOqua-Lect4, scaffold 1: 1.30 Mb), and an additional homolog near the same region. These likely encode C-type lectin-like proteins capable of binding glycans or lipid moieties on host cell membranes. Such lectins may facilitate cell agglutination or complement modulation. The proximity of LOqua-Lect1 and LOqua-Lect3 on scaffold 7 suggests a history of tandem gene duplication in some lipopolysaccharide-binding proteins. Two lipocalin homologs were recovered which include LOqua-Lipcl3 (scaffold 38: 4.64 Mb) and LOqua-Lipcl1 (scaffold 51: 1.89 to 2.76 Mb). These small ligand-binding proteins, homologous to odorant-binding and biliverdin-binding proteins, may act as scavengers for small hydrophobic molecules, such as heme or prostaglandins, thereby modulating oxidative or inflammatory responses in the host. The extended genomic interval of LOqua-Lipcl1 may represent either a large intron-rich structure or a local paralog cluster. Two compact antimicrobial loci were mapped which include attacin E (LOqua-Def3, scaffold 15: 0.22 Mb) and cecropin 6 (LOqua-Def4, scaffold 53: 0.29 Mb). Both are short, single-exon peptides typical of lepidopteran AMPs and likely contribute to the antimicrobial or cytolytic environment created at the sting site. These genes might serve defensive functions within the caterpillar's hemolymph but could also exacerbate local tissue damage upon envenomation. A defensin-like precursor (LOqua-PI9, AY829837) was not confidently mapped to the *L. casanarensis* genome. Partial alignments indicate additional Kazal-type and trypsin-inhibitor-like domains, though not all could be placed with high confidence. These inhibitors likely cooperate with serpins and cystatin to regulate protease activity within the venom gland. Similarly, a phospholipase A2 (PLA2) homolog was detected at the transcript level but lacked a clear genomic match. Our analyses were guided by the transcriptomic catalog of (Veiga et al. 2005), which, to our knowledge, was compiled prior to the characterization, sequencing, and identification of the *L. obliqua* venom procoagulant factor Losac (Alvarez-Flores et al. 2006, Alvarez-Flores et al. 2011). Losac, a hemolin (an immunoglobulin-like protein), functions as an activator of the human coagulation factor X (Alvarez-Flores et al. 2011). Both Losac and the procoagulant lipocalin Lopap are implicated in the consumption coagulopathy associated with *L. obliqua* envenomation (Alvarez-Flores et al. 2006, Alvarez-Flores et al. 2011). Neither Losac nor members of the hemolin protein family, which have also been reported to play important roles in insect immune modulation and tissue repair (Wojda et al. 2020), are present in the reference transcriptome (Veiga et al. 2005), and therefore are absent from our results. Given the significance of Losac in *L. obliqua* envenoming and the involvement of hemolins in immune modulation, future research should investigate the presence of hemolin homologs in the *L. casanarensis* genome and assess their expression in venom-associated tissues.

**Table 1.** Mapping of *L. obliqua* venom cDNAs to the *L. casanarensis* genome.

Sequence name	GenBank ID	Match reported in Veiga et al. (2005)	Match to *Lonomia casanarensis* genome (Scaffold: Start, End)	
**LOqua-CysPep1**	AY829805	Cathepsin L-like protease	Scaffold_30 : 237605, 237905	
**LOqua-CysPep2**	AY829838	Cathepsin B-like cysteine proteinase	Scaffold_7 : 2998510, 3043173	
**LOqua-Cyst1**	AY829806	L-cystatin precursor	Scaffold_32 : 2840512, 2842889	
**LOqua-Def3**	AY829840	Attacin E precursor	Scaffold_15 : 225469, 226500	
**LOqua-Def4**	AY829848	Antimicrobial peptide cecropin 6	Scaffold_53 : 299003, 299700	
**LOqua-Lect1**	AY829822	Lipopolysaccharide binding protein	Scaffold_7: 11445649, 11451586	
**LOqua-Lect3**	AY829836	Lipopolysaccharide binding protein	Scaffold_7: 11450799, 11457067	
**LOqua-Lect4**	AY829849	Unknown	Scaffold_1 : 1307642, 1308489	
**LOqua-Lipcl1**	AY829833	Biliverdin binding protein-II	Scaffold_51 : 1893523, 2760584	
**LOqua-Lipcl3**	AY829856	Odorant-binding protein	Scaffold_38 : 4641331, 4644045	
**LOqua-PI9**	AY829837	Plant defensin precursor	Unmapped/no scaffold match found	
**LOqua-Serp1**	AY829814	Serpin	Scaffold_8 : 1863761, 1874000	
**LOqua-Serp3**	AY829816	Serpin	Scaffold_12 : 7397274, 7398610	
**LOqua-Serp6**	AY829847	Serpin	Scaffold_15 : 6425965, 6426900	
**LOqua-SP3**	AY829820	30kP protease A precursor	Scaffold_9 : 10066878, 10070500	
**LOqua-SP4**	AY829821	Trypsin-like serine protease	Scaffold_5 : 2410284, 2422807	
**LOqua-SP5**	AY829842	Unknown	Scaffold_21 : 2117824, 2119600	
**LOqua-SP7**	AY829841	Trypsin-like serine protease	Scaffold_34 : 3808701, 3812800	

In summary, venom-related genes are broadly dispersed across the *L. casanarensis* genome, with occasional microclusters such as the lectins on scaffold 7 (Supplementary Fig. 1). Despite structural divergence, the preservation of core protease and inhibitor genes remains and suggests a protease-rich venom cocktail which altogether produces the hemorrhagic outcome.

## Supplementary Material

jkag113_Supplementary_Data

## Data Availability

Genome assemblies are archived on NCBI Sequence Read Archive and are available under Bioproject number PRJNA1338714 and PRJNA1440448 (https://www.ncbi.nlm.nih.gov/bioproject/PRJNA1338714), Biosample number SAMN52583689, accession number JBWLKR000000000, and SRA number SRR36131380. All the scripts used for the generation of the genome assembly and the downstream data analyses are available on the GitHub link: https://github.com/chaturvedi-lab/Lonomia_genome_assembly.git All output files associated with the genome annotation and relevant bioinformatics pipeline and genome assemblies are available on this Zenodo link: https://doi.org/10.5281/zenodo.19134648. Supplemental material available at G3 online.

## References

[jkag113-B1] Alvarez-Flores MP, Fritzen M, Reis CV, Chudzinski-Tavassi AM. 2006. Losac, a factor X activator from *Lonomia obliqua* bristle extract: its role in the pathophysiological mechanisms and cell survival. Biochem Biophys Res Commun. 343:1216–1223. 10.1016/j.bbrc.2006.03.068. 16597435

[jkag113-B2] Alvarez-Flores MP et al 2011. Losac, the first hemolin that exhibits procoagulant activity through selective factor X proteolytic activation. J Biol Chem. 286:6918–6928. 10.1074/jbc.M110.167718. 21177860 PMC3044947

[jkag113-B3] Arocha-Piñango CL et al 1992. Six new cases of a caterpillar-induced bleeding syndrome. Thromb Haemost. 67:402–407. 10.1055/s-0038-1648460. 1378651

[jkag113-B4] Bernt M et al 2013. MITOS: improved de novo metazoan mitochondrial genome annotation. Mol Phylogenet Evol. 69:313–319. 10.1016/j.ympev.2012.08.023. 22982435

[jkag113-B5] Carrijo-Carvalho LC, Chudzinski-Tavassi AM. 2007. The venom of the *Lonomia* caterpillar: an overview. Toxicon. 49:741–757. 10.1016/j.toxicon.2006.11.033. 17320134

[jkag113-B6] Challis R, Richards E, Rajan J, Cochrane G, Blaxter M. 2020. BlobToolKit: interactive quality assessment of genome assemblies. G3 (Bethesda). 10:1361–1374. 10.1534/g3.119.400908. 32071071 PMC7144090

[jkag113-B7] Cheng H et al 2022. Haplotype-resolved assembly of diploid genomes without parental data. Nat Biotechnol. 40:1332–1335. 10.1038/s41587-022-01261-x. 35332338 PMC9464699

[jkag113-B8] Coppens B . 2016. Kweek en beschrijving van de levensstadia van *Lonomia electra* in gevangenschap (Lepidoptera: Saturniidae). Entomolog Ber. 76:213–217. https://natuurtijdschriften.nl/pub/1011472.

[jkag113-B9] Da Silva WD et al 1996. Short communication. Development of an antivenom against toxins of *Lonomia obliqua* caterpillars. Toxicon. 34:1045–1049. 10.1016/0041-0101(96)00052-9. 8896196

[jkag113-B10] Favalesso MM, Cuervo PF, Casafús MG, Guimarães ATB, Peichoto ME. 2021. *Lonomia* envenomation in Brazil: an epidemiological overview for the period 2007–2018. Trans R Soc Trop Med Hyg. 115:9–19. 10.1093/trstmh/traa051. 32945864

[jkag113-B11] Gamborgi GP, Metcalf EB, Barros EJG. 2006. Acute renal failure provoked by toxin from caterpillars of the species *Lonomia obliqua*. Toxicon. 47:68–74. 10.1016/j.toxicon.2005.09.012. 16310819

[jkag113-B12] Ghurye J, Pop M, Koren S, Bickhart D, Chin C-S. 2017. Scaffolding of long read assemblies using long range contact information. BMC Genomics. 18:527. 10.1186/s12864-017-3879-z. 28701198 PMC5508778

[jkag113-B13] Gonçalves LRC, Sousa-e-Silva MCC, Tomy SC, Sano-Martins IS. 2007. Efficacy of serum therapy on the treatment of rats experimentally envenomed by bristle extract of the caterpillar *Lonomia obliqua*: comparison with epsilon-aminocaproic acid therapy. Toxicon. 50:349–356. 10.1016/j.toxicon.2007.04.004. 17537473

[jkag113-B14] González C et al 2023. Deadly and venomous *Lonomia caterpillars* are more than the two usual suspects. PLoS Negl Trop Dis. 17:e0011063. 10.1371/journal.pntd.0011063. 36821543 PMC9949635

[jkag113-B15] Gouet P, Robert X, Courcelle E. 2003. ESPript/ENDscript: extracting and rendering sequence and 3D information from atomic structures of proteins. Nucleic Acids Res. 31:3320–3323. 10.1093/nar/gkg556. 12824317 PMC168963

[jkag113-B16] Guerrero B et al 2001. Thrombolytic effect of lonomin V in a rabbit jugular vein thrombosis model. Blood Coagul Fibrinolysis. 12:521–529. 10.1097/00001721-200110000-00003. 11685039

[jkag113-B17] Guerrero B et al 2011. The effects of Lonomin V, a toxin from the caterpillar (*Lonomia achelous*), on hemostasis parameters as measured by platelet function. Toxicon. 58:293–303. 10.1016/j.toxicon.2011.07.003. 21820001

[jkag113-B18] Gurevich A, Saveliev V, Vyahhi N, Tesler G. 2013. QUAST: quality assessment tool for genome assemblies. Bioinformatics. 29:1072–1075. 10.1093/bioinformatics/btt086. 23422339 PMC3624806

[jkag113-B19] Huang N, Li H. 2023. Compleasm: a faster and more accurate reimplementation of BUSCO. Bioinformatics. 39:btad595. 10.1093/bioinformatics/btad595. 37758247 PMC10558035

[jkag113-B41] Kalyaanamoorthy S et al 2017. ModelFinder: fast model selection for accurate phylogenetic estimates. Nat Methods. 14:587–589. 10.1038/nmeth.4285. 28481363 PMC5453245

[jkag113-B20] Katoh K, Standley DM. 2013. MAFFT multiple sequence alignment software version 7: improvements in performance and usability. Mol Biol Evol. 30:772–780. 10.1093/molbev/mst010. 23329690 PMC3603318

[jkag113-B21] Li H . 2018. Minimap2: pairwise alignment for nucleotide sequences. Bioinformatics. 34:3094–3100. 10.1093/bioinformatics/bty191. 29750242 PMC6137996

[jkag113-B22] Li H . 2021. New strategies to improve minimap2 alignment accuracy. Bioinformatics. 37:4572–4574. 10.1093/bioinformatics/btab705. 34623391 PMC8652018

[jkag113-B23] Minh BQ et al 2020. IQ-TREE 2: new models and efficient methods for phylogenetic inference in the genomic era. Mol Biol Evol. 37:1530–1534. 10.1093/molbev/msaa015. 32011700 PMC7182206

[jkag113-B24] Perrier C et al 2025. Transposable element accumulation drives genome size increase in *Hylesia metabus* (Lepidoptera: Saturniidae), an urticating moth species from South America. J Hered. 116:344–353. 10.1093/jhered/esae069. 39556100 PMC12130439

[jkag113-B25] Pineda D, Amarillo A, Becerra J, Montenegro G. 2001. Síndrome hemorrágico por contacto con orugas del género *Lonomia* (Saturniidae) en Casanare, Colombia: informe de dos casos. Biomédica. 21:328–332. 10.7705/biomedica.v21i4.1125.

[jkag113-B26] Pinto AFM, Berger M, Reck J Jr, Terra RMS, Guimarães JA. 2010. *Lonomia obliqua* venom: in vivo effects and molecular aspects associated with the hemorrhagic syndrome. Toxicon. 56:1103–1112. 10.1016/j.toxicon.2010.01.013. 20114060

[jkag113-B27] Ranallo-Benavidez TR, Jaron KS, Schatz MC. 2020. GenomeScope 2.0 and Smudgeplot for reference-free profiling of polyploid genomes. Nat Commun. 11:1432. 10.1038/s41467-020-14998-3. 32188846 PMC7080791

[jkag113-B28] Rhie A, Walenz BP, Koren S, Phillippy AM. 2020. Merqury: reference-free quality, completeness, and phasing assessment for genome assemblies. Genome Biol. 21:245. 10.1186/s13059-020-02134-9. 32928274 PMC7488777

[jkag113-B29] Riva HG, Amarillo-S AR. 2023. A systematic review of the bioprospecting potential of *Lonomia* spp. (Lepidoptera: Saturniidae). Toxin Rev. 42:583–598. 10.1080/15569543.2023.2204348.

[jkag113-B30] Rougerie R et al 2022. Phylogenomics illuminates the evolutionary history of wild silkmoths in space and time (Lepidoptera: Saturniidae). [preprint]. bioRxiv 486224. 10.1101/2022.03.29.486224. 42500966

[jkag113-B31] Sano-Martins IS, González C, Anjos IV, Díaz J, Gonçalves LRC. 2018. Effectiveness of *Lonomia* antivenom in recovery from the coagulopathy induced by *Lonomia orientoandensis* and *Lonomia casanarensis* caterpillars in rats. PLoS Negl Trop Dis. 12:e0006721. 10.1371/journal.pntd.0006721. 30114211 PMC6112677

[jkag113-B32] Sim SB, Corpuz RL, Simmonds TJ, Geib SM. 2022. HiFiAdapterFilt, a memory efficient read processing pipeline, prevents occurrence of adapter sequence in PacBio HiFi reads and their negative impacts on genome assembly. BMC Genomics. 23:157. 10.1186/s12864-022-08375-1. 35193521 PMC8864876

[jkag113-B33] Simão FA, Waterhouse RM, Ioannidis P, Kriventseva EV, Zdobnov EM. 2015. BUSCO: assessing genome assembly and annotation completeness with single-copy orthologs. Bioinformatics. 31:3210–3212. 10.1093/bioinformatics/btv351. 26059717

[jkag113-B34] Skojec C, Godfrey RK, Kawahara AY. 2024. Long read genome assembly of *Automeris io* (*Lepidoptera: Saturniidae*) an emerging model for the evolution of deimatic displays. G3 (Bethesda). 14:jkae048. 10.1093/g3journal/jkad292. 38324397 PMC10917498

[jkag113-B35] Spadacci-Morena DD, Soares MAM, Moraes RHP, Sano-Martins IS, Sciani JM. 2016. The urticating apparatus in the caterpillar of *Lonomia obliqua* (Lepidoptera: Saturniidae). Toxicon. 119:218–224. 10.1016/j.toxicon.2016.06.008. 27319295

[jkag113-B42] Toro-Vargas DM, González C, Rougerie R, Amarillo-Suárez AR. 2023. Characterization of morphological and biological aspects of venomous caterpillars of the genus Lonomia Walker (Lepidoptera: Saturniidae) in Colombia. Plos one 18:p.e0285010. 10.1371/journal.pone.0285010. PMC10231817 37256837

[jkag113-B36] Uliano-Silva M et al 2023. MitoHiFi: a python pipeline for mitochondrial genome assembly from PacBio high fidelity reads. BMC Bioinformatics. 24:288. 10.1186/s12859-023-05385-y. 37464285 PMC10354987

[jkag113-B37] Veiga ABG, Ribeiro JMC, Guimarães JA, Francischetti IMB. 2005. A catalog for the transcripts from the venomous structures of the caterpillar *Lonomia obliqua*: identification of the proteins potentially involved in the coagulation disorder and hemorrhagic syndrome. Gene. 355:11–27. 10.1016/j.gene.2005.05.002. 16023793 PMC2909119

[jkag113-B38] Wojda I, Cytryńska M, Zdybicka-Barabas A, Kordaczuk J. 2020. Insect defense proteins and peptides. In: Hoeger U, Harris J, editors. Vertebrate and invertebrate respiratory proteins, lipoproteins and other body fluid proteins. Vol. 94. Subcellular Biochemistry. p. 75–108. 10.1007/978-3-030-41769-7_4 32189297

[jkag113-B39] Zhang C, Rabiee M, Sayyari E, Mirarab S. 2018. ASTRAL-III: polynomial time species tree reconstruction from partially resolved gene trees. BMC Bioinformatics. 19:153. 10.1186/s12859-018-2129-y. 29745866 PMC5998893

