# Supplementary material for: Genome assembly and venom gene mapping in the medically important moth Lonomia casanarensis (Saturniidae: Hemileucinae)
Source: G3 (Bethesda). 2026 May 13;16(7):jkag113. doi: 10.1093/g3journal/jkag113 (PMC13334177; doi:10.1093/g3journal/jkag113)
Supplement: jkag113_Supplementary_Data [file jkag113_supplementary_data.zip › Supplemental_Material_G3-2025-406412.docx]

## Supplementary Tables

| Assembly | Haplotype 1 | | Haplotype 2 |
| --- | --- | --- | --- |
| # contigs (>= 0 bp) | 118 | 131 | |
| # contigs (>= 1000 bp) | 118 | 131 | |
| # contigs (>= 5000 bp) | 118 | 131 | |
| # contigs (>= 10000 bp) | 118 | 131 | |
| # contigs (>= 25000 bp) | 117 | 131 | |
| # contigs (>= 50000 bp) | 106 | 130 | |
| Total length (>= 0 bp) | 483832926 | 479325004 | |
| Total length (>= 1000 bp) | 483832926 | 479325004 | |
| Total length (>= 5000 bp) | 483832926 | 479325004 | |
| Total length (>= 10000 bp) | 483832926 | 479325004 | |
| Total length (>= 25000 bp) | 483810889 | 479325004 | |
| Total length (>= 50000 bp) | 483402937 | 479281430 | |
| # contigs | 118 | 131 | |
| Largest contig | 19371879 | 15977336 | |
| Total length | 483832926 | 479325004 | |
| GC (%) | 35.97 | 35.95 | |
| N50 | 7192505 | 7509013 | |
| N90 | 2323681 | 1645082 | |
| auN | 7704468.4 | 7748092.6 | |
| L50 | 24 | 22 | |
| L90 | 65 | 71 | |
| # N's per 100 kbp | 3.72 | 0 | |

**Table S1. QUAST assembly statistics as generated for both haplotypes.**

| Substitution Model | Count | Percentage |
| --- | --- | --- |
| JTT+G4 | 311 | 27.92% |
| JTT+I | 126 | 11.31% |
| JTT+R2 | 90 | 8.08% |
| JTTDCMut+G4 | 63 | 5.66% |
| LG+I | 56 | 5.03% |
| LG+G4 | 56 | 5.03% |
| JTT+I+G4 | 39 | 3.50% |
| HIVb+G4 | 26 | 2.33% |
| JTTDCMut+I | 24 | 2.15% |
| LG | 23 | 2.06% |
| JTTDCMut+I+G4 | 23 | 2.06% |
| JTT+F+G4 | 21 | 1.88% |
| LG+R2 | 19 | 1.71% |
| JTT+R3 | 19 | 1.71% |
| JTTDCMut+R2 | 18 | 1.62% |
| FLU+G4 | 18 | 1.62% |
| mtMet+F+G4 | 13 | 1.17% |
| JTT+F+I+G4 | 13 | 1.17% |
| Dayhoff+I | 13 | 1.17% |
| mtZOA+G4 | 12 | 1.08% |
| LG+I+G4 | 10 | 0.90% |
| Dayhoff+G4 | 10 | 0.90% |
| HIVb+I | 9 | 0.81% |
| Dayhoff | 9 | 0.81% |
| mtZOA+I | 8 | 0.72% |
| JTTDCMut+R3 | 8 | 0.72% |
| HIVb+F+G4 | 8 | 0.72% |
| FLU+I | 8 | 0.72% |
| mtMet+F+I+G4 | 7 | 0.63% |
| JTT+F+R3 | 7 | 0.63% |
| JTT | 7 | 0.63% |
| cpREV+G4 | 5 | 0.45% |
| WAG+G4 | 4 | 0.36% |
| HIVb+F+I | 4 | 0.36% |
| Blosum62 | 4 | 0.36% |
| WAG | 3 | 0.27% |
| mtMet+F+R2 | 3 | 0.27% |
| JTT+F+R4 | 3 | 0.27% |
| JTTDCMut+F+G4 | 3 | 0.27% |
| HIVb+R2 | 3 | 0.27% |
| HIVb+F+R3 | 3 | 0.27% |
| HIVb+F+I+G4 | 3 | 0.27% |
| Dayhoff+R2 | 3 | 0.27% |
| cpREV+I | 3 | 0.27% |
| Blosum62+I+G4 | 3 | 0.27% |
| Other (models with n < 3) | 69 | 6.19% |
| Total | 1,114 |  |

**Table S2. Summary of substitution models selected by IQ-TREE for all gene alignments used in the nuclear species tree.**

| **Metric** | **Value** |
| --- | --- |
| Predicted protein-coding genes | 33191 |
| Predicted transcripts | 37088 |
| Monoexonic transcripts | 7097 |
| Monoexonic transcripts (%) | 19.14 |
| Multiexonic transcripts | 29991 |
| Multiexonic transcripts (%) | 80.86 |
| Mean exons per transcript | 5.82 |
| Mean gene length (bp) | 7127.1 |
| Mean exon length (bp) | 227.9 |
| Mean intron length (bp) | 1279.4 |
| Mean CDS length (bp) | 1327.1 |
| BUSCO complete (%) | 97.7 |
| BUSCO single-copy (%) | 93.5 |
| BUSCO duplicated (%) | 4.2 |
| BUSCO fragmented (%) | 0.7 |
| BUSCO missing (%) | 1.6 |
| Proteins with InterProScan hits | 32013 |
| Proteins with InterProScan hits (%) | 86.32 |
| Unique InterPro entries | 12620 |
| Unique Pfam entries | 5921 |
| Proteins with eggNOG annotation | 30815 |
| Proteins with eggNOG annotation (%) | 83.09 |
| Proteins with KEGG pathways (eggNOG) | 11279 |

**Table S3 Summary of metrics generated from genome annotations using BRAKER3, InterProScan and EggNOG.**

# Supplementary Figures


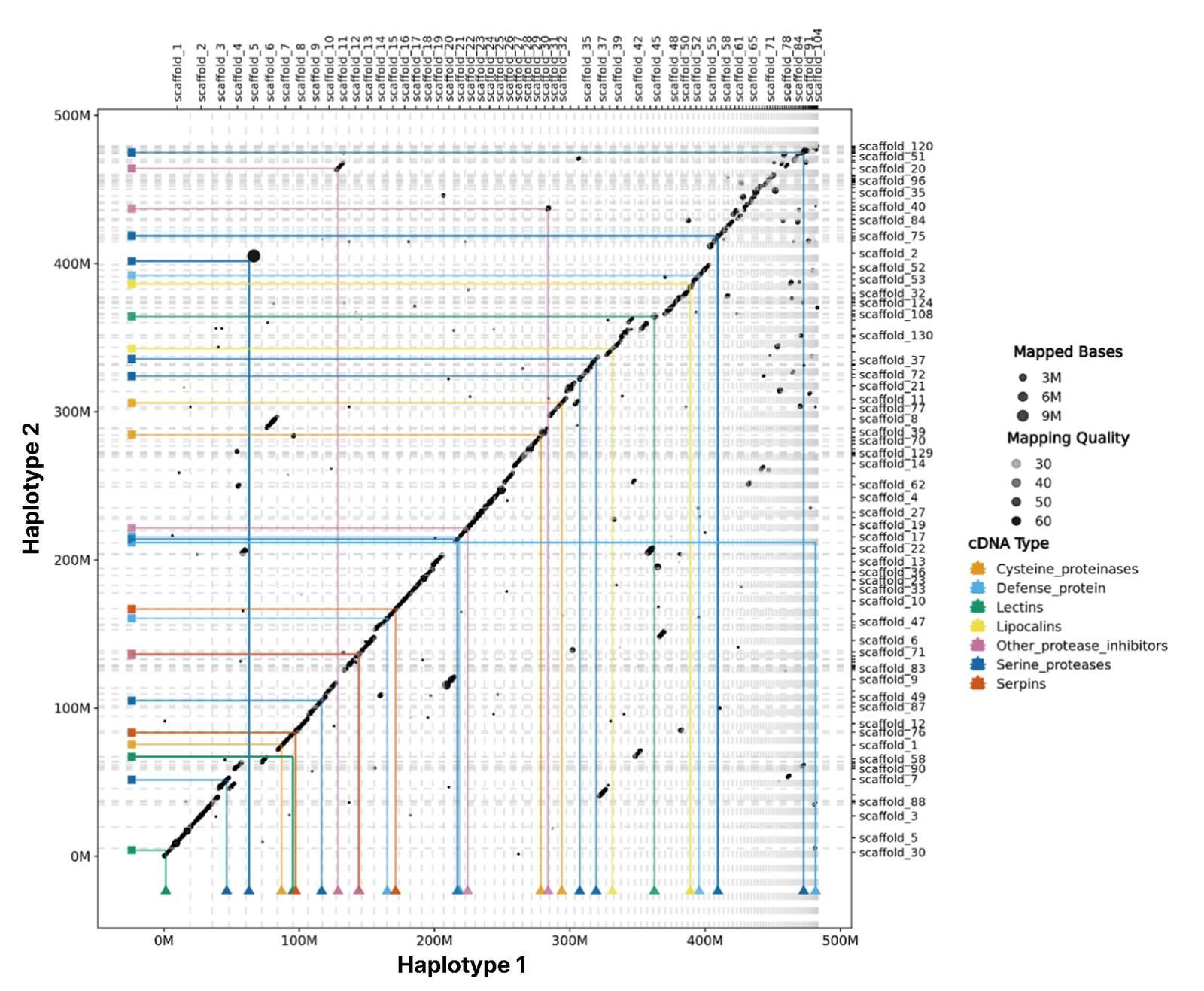


**Figure S1**: Mapping between the two haplotypes of the *L. casanarensis* genome assembly.

##

##
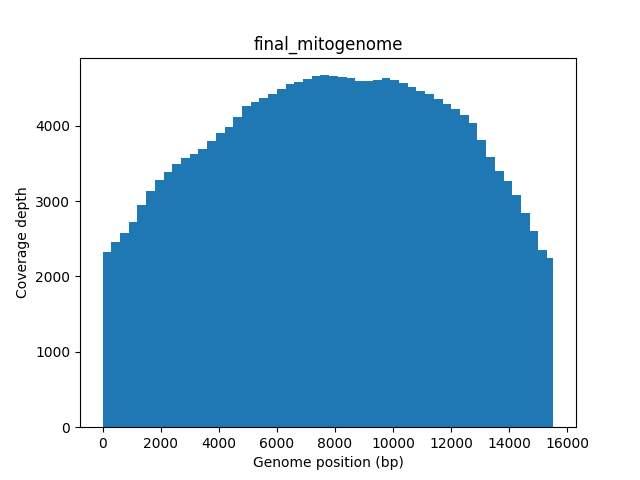


**Figure S2:** Coverage of PacBio reads along the mitochondrial genome of  *L. casanarensis*.

##

##
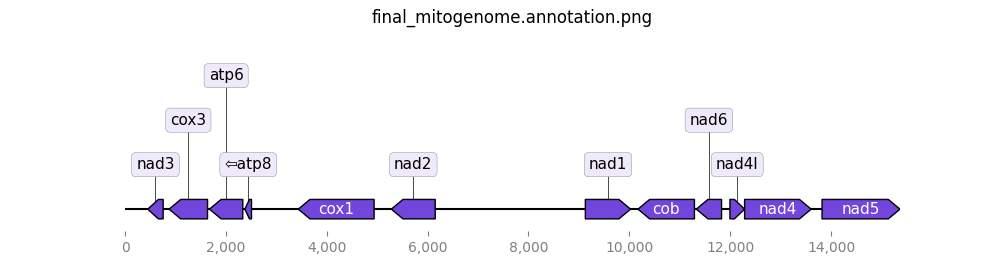


**Figure S3:** Annotation of mitochondrial genes along the mitochondrial genome of *L. casanarensis*.

## 
