# Supplementary figures and images for: Genome assembly and venom gene mapping in the medically important moth Lonomia casanarensis (Saturniidae: Hemileucinae)
Source: G3 (Bethesda). 2026 May 13;16(7):jkag113. doi: 10.1093/g3journal/jkag113 (PMC13334177; doi:10.1093/g3journal/jkag113)

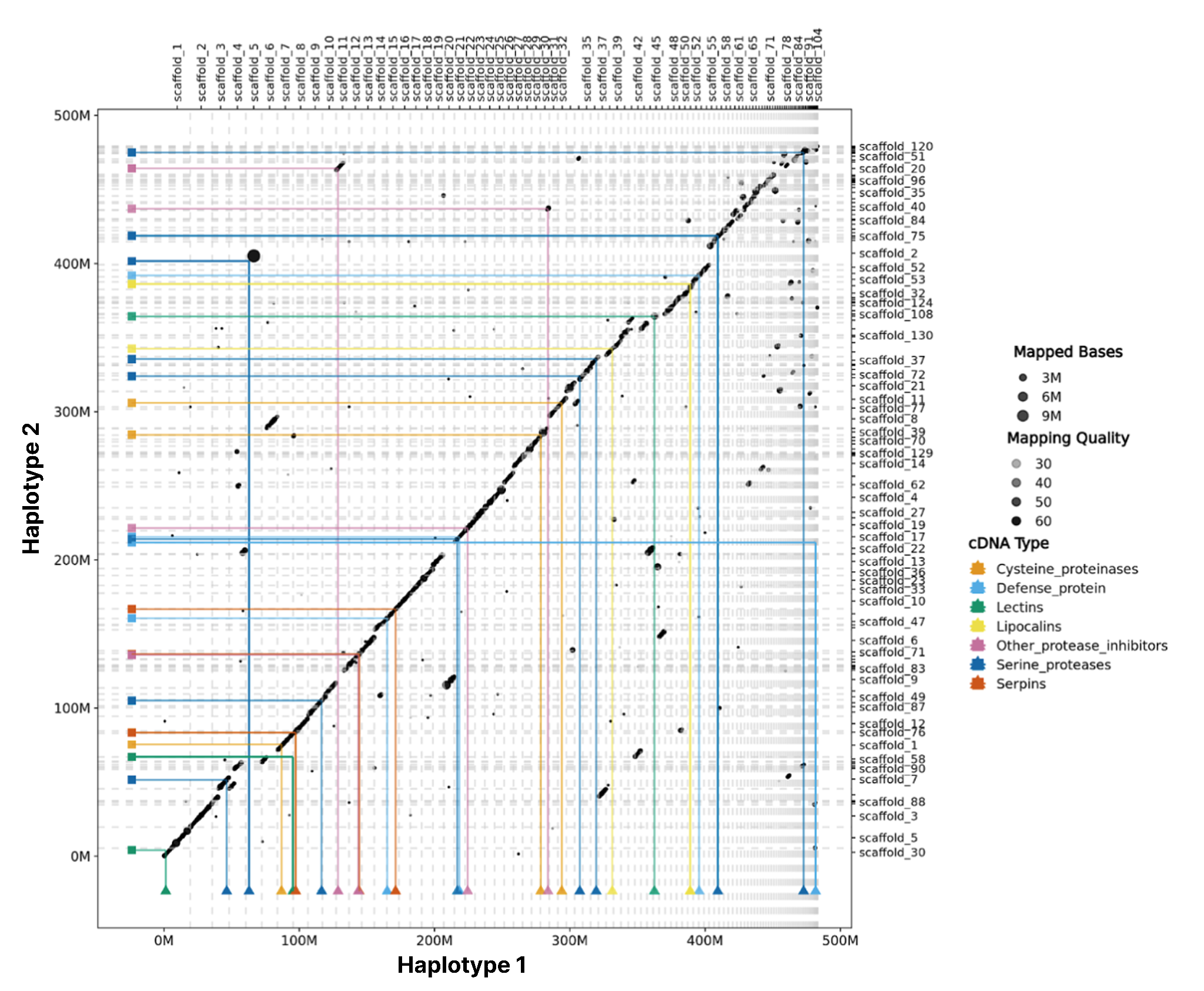

Supplement: jkag113_Supplementary_Data [file jkag113_supplementary_data.zip › FigS01_G3-2025-406412.png]

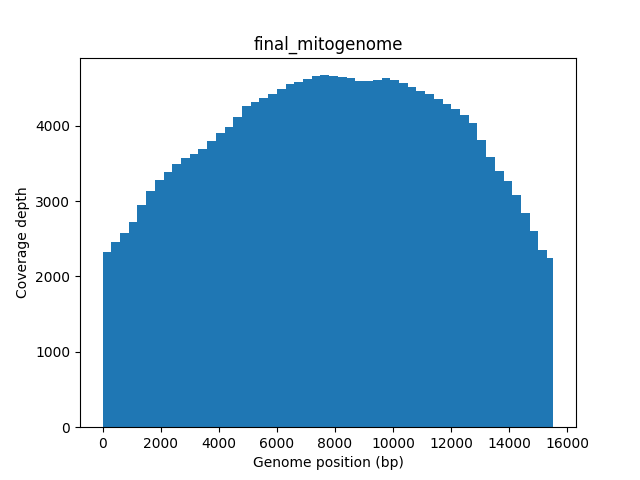

Supplement: jkag113_Supplementary_Data [file jkag113_supplementary_data.zip › FigS02_G3-2025-406412.png]

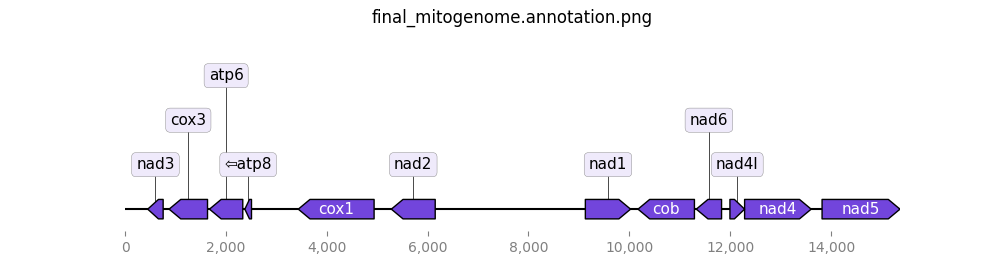

Supplement: jkag113_Supplementary_Data [file jkag113_supplementary_data.zip › FigS03_G3-2025-406412.png]

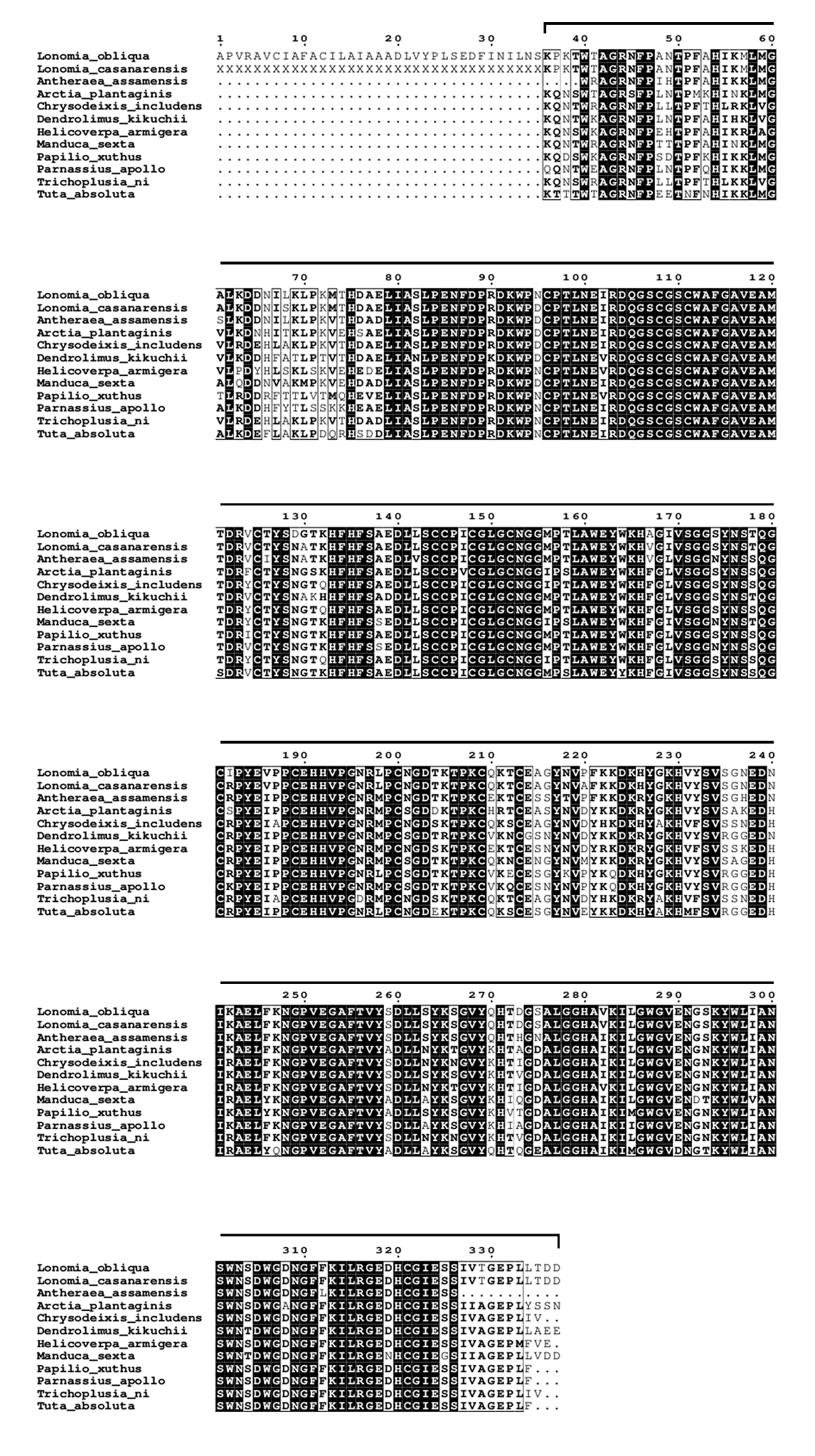

Supplement: jkag113_Supplementary_Data [file jkag113_supplementary_data.zip › FigS04_G3-2025-406412.png]

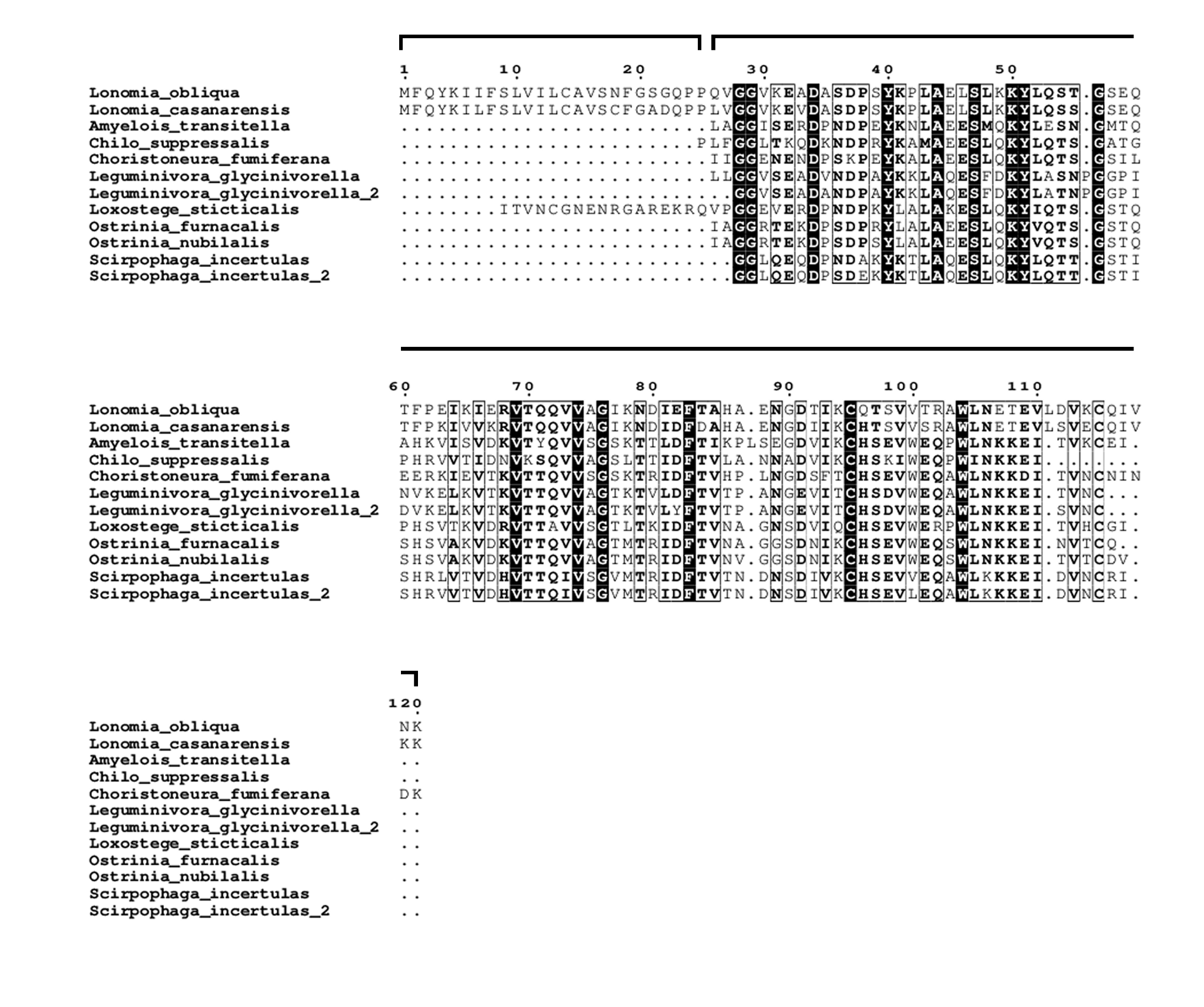

Supplement: jkag113_Supplementary_Data [file jkag113_supplementary_data.zip › FigS05_G3-2025-406412.png]

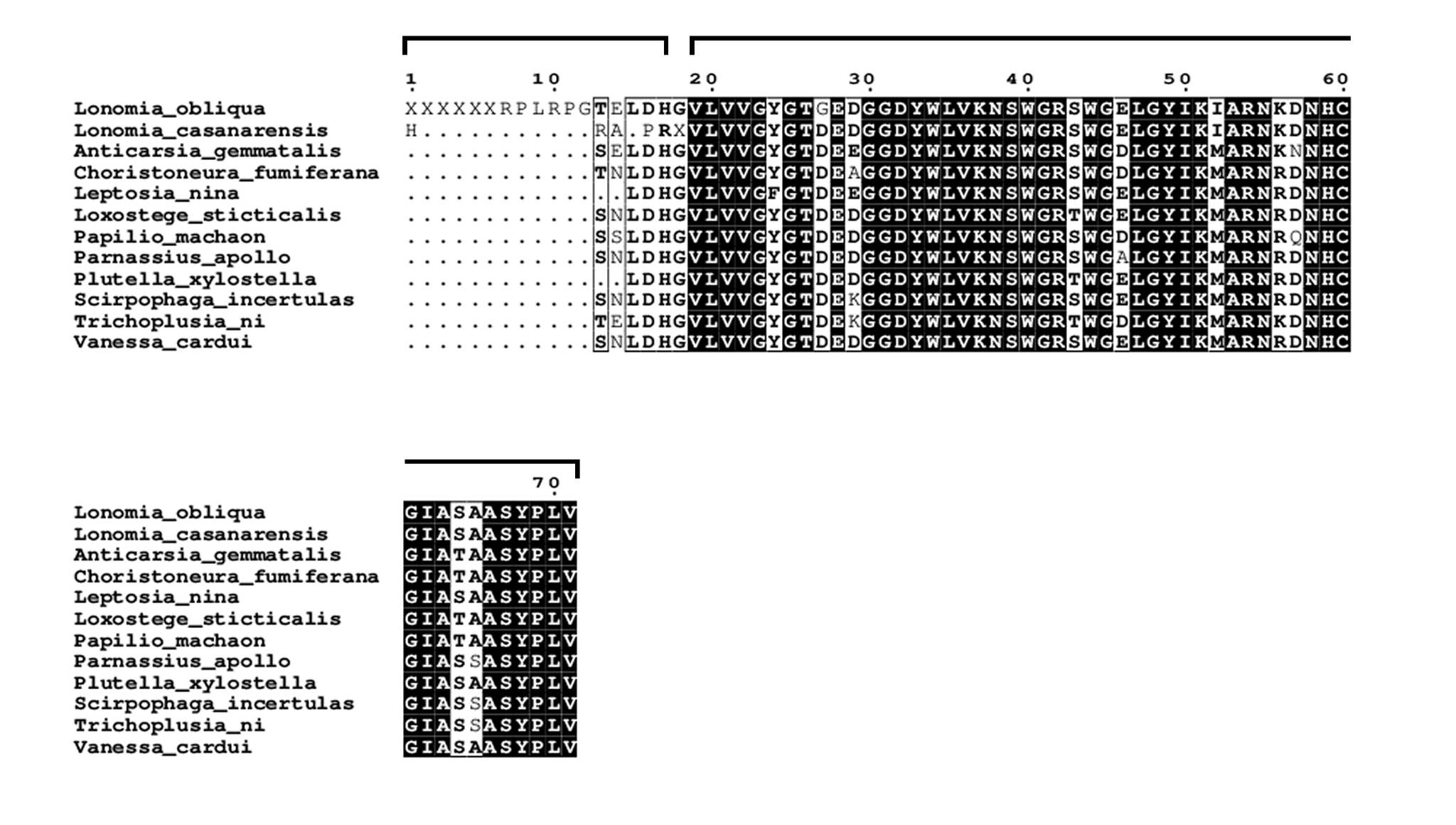

Supplement: jkag113_Supplementary_Data [file jkag113_supplementary_data.zip › FigS06_G3-2025-406412.png]

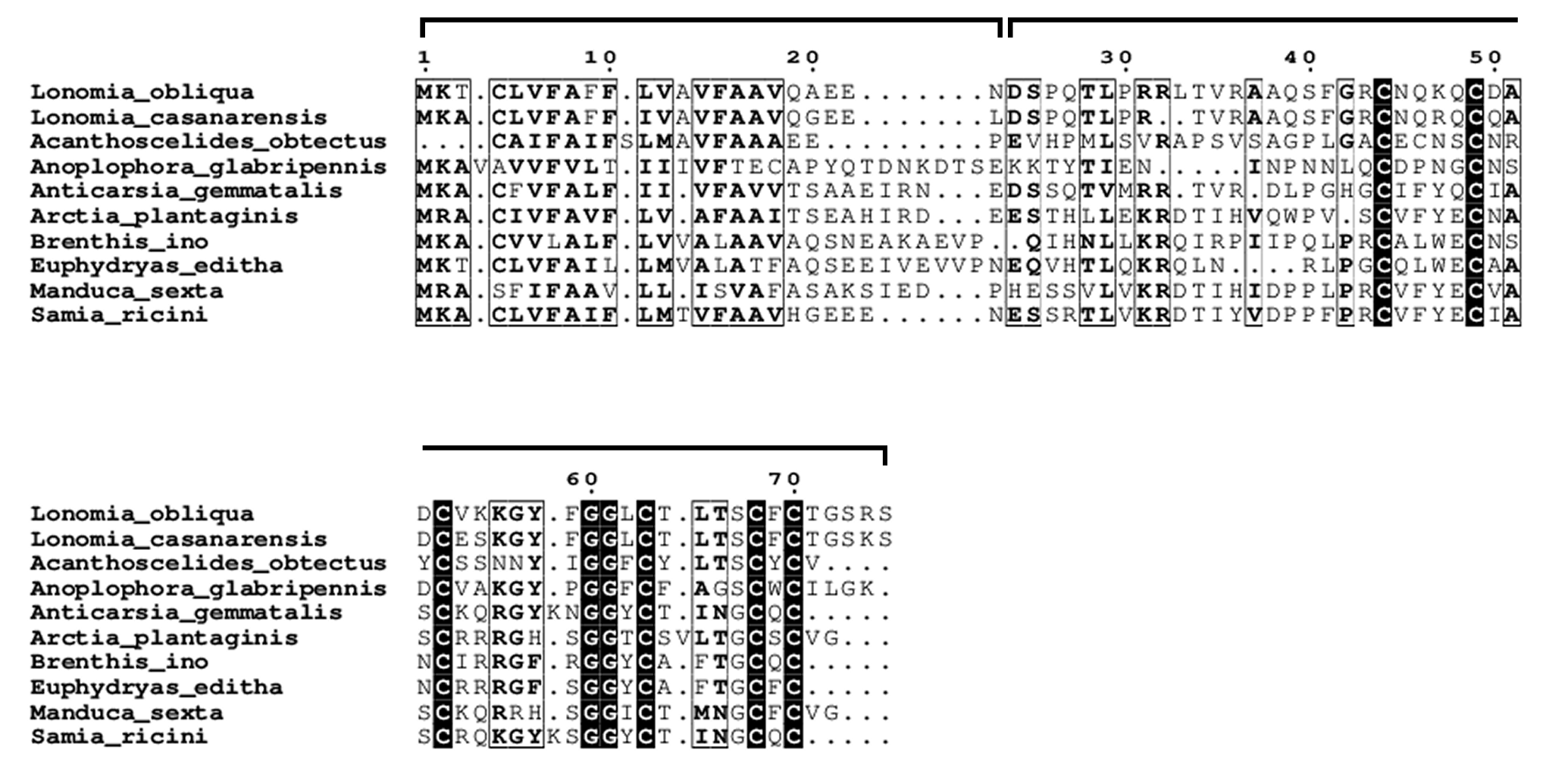

Supplement: jkag113_Supplementary_Data [file jkag113_supplementary_data.zip › FigS07_G3-2025-406412.png]

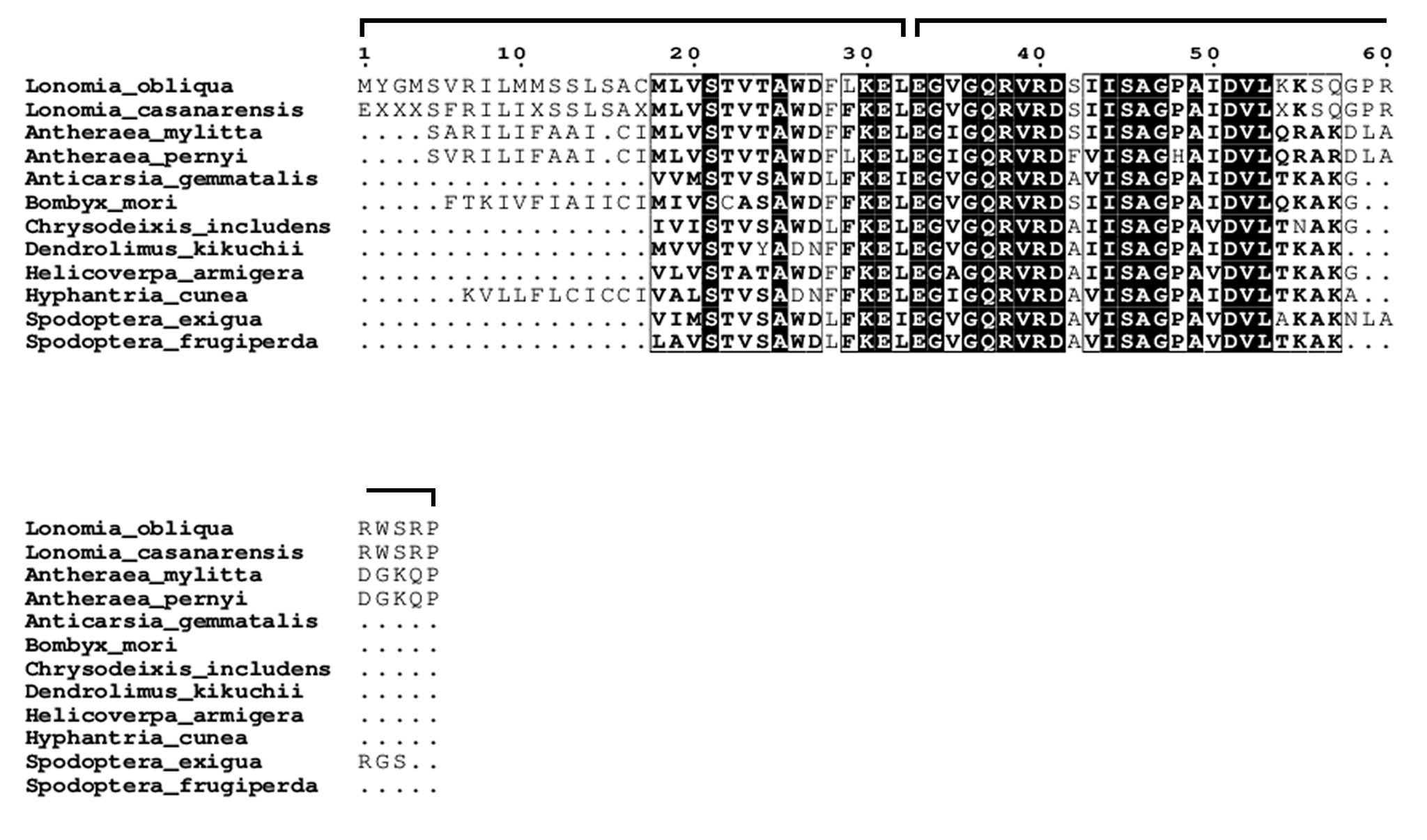

Supplement: jkag113_Supplementary_Data [file jkag113_supplementary_data.zip › FigS08_G3-2025-406412.png]

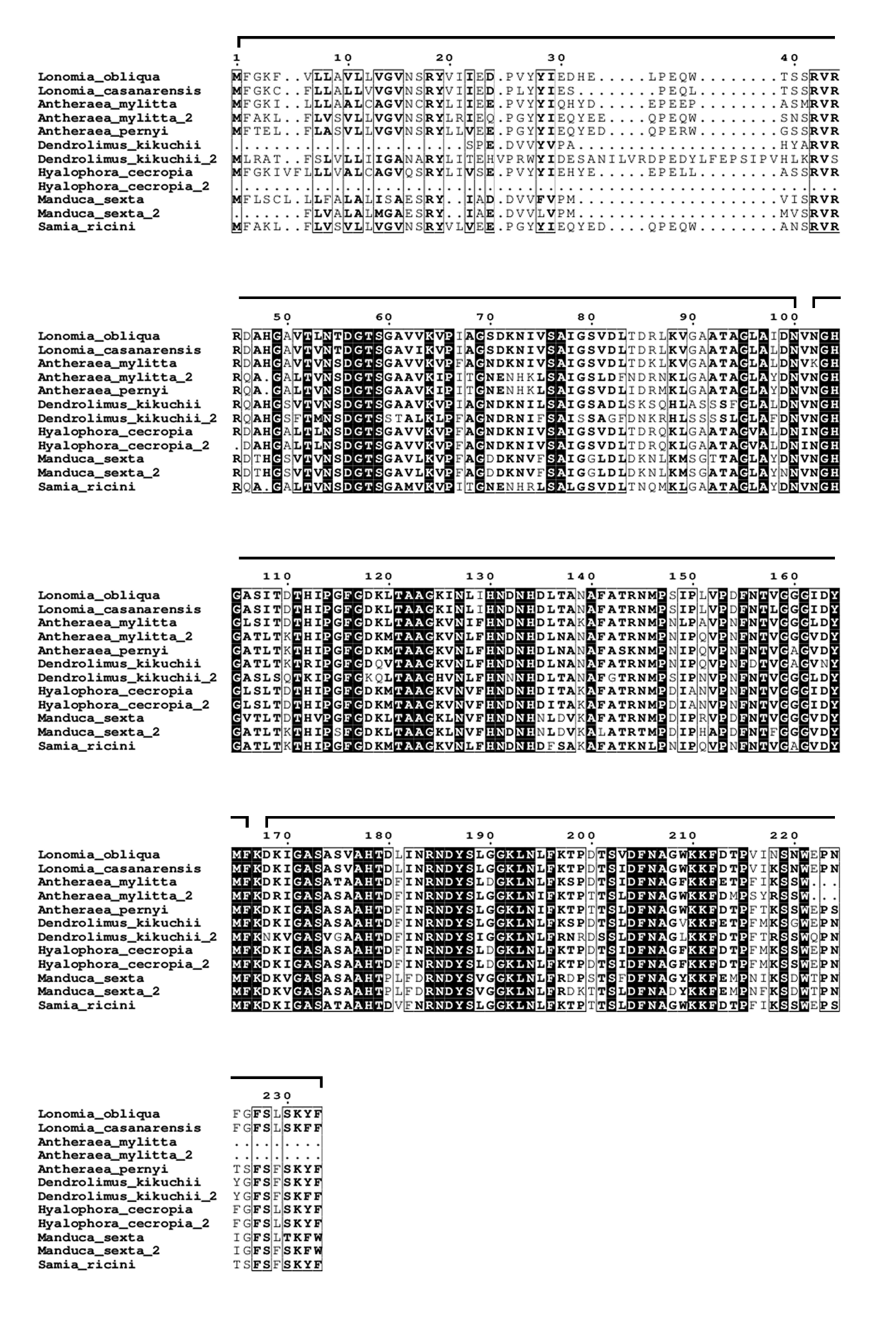

Supplement: jkag113_Supplementary_Data [file jkag113_supplementary_data.zip › FigS09_G3-2025-406412.png]

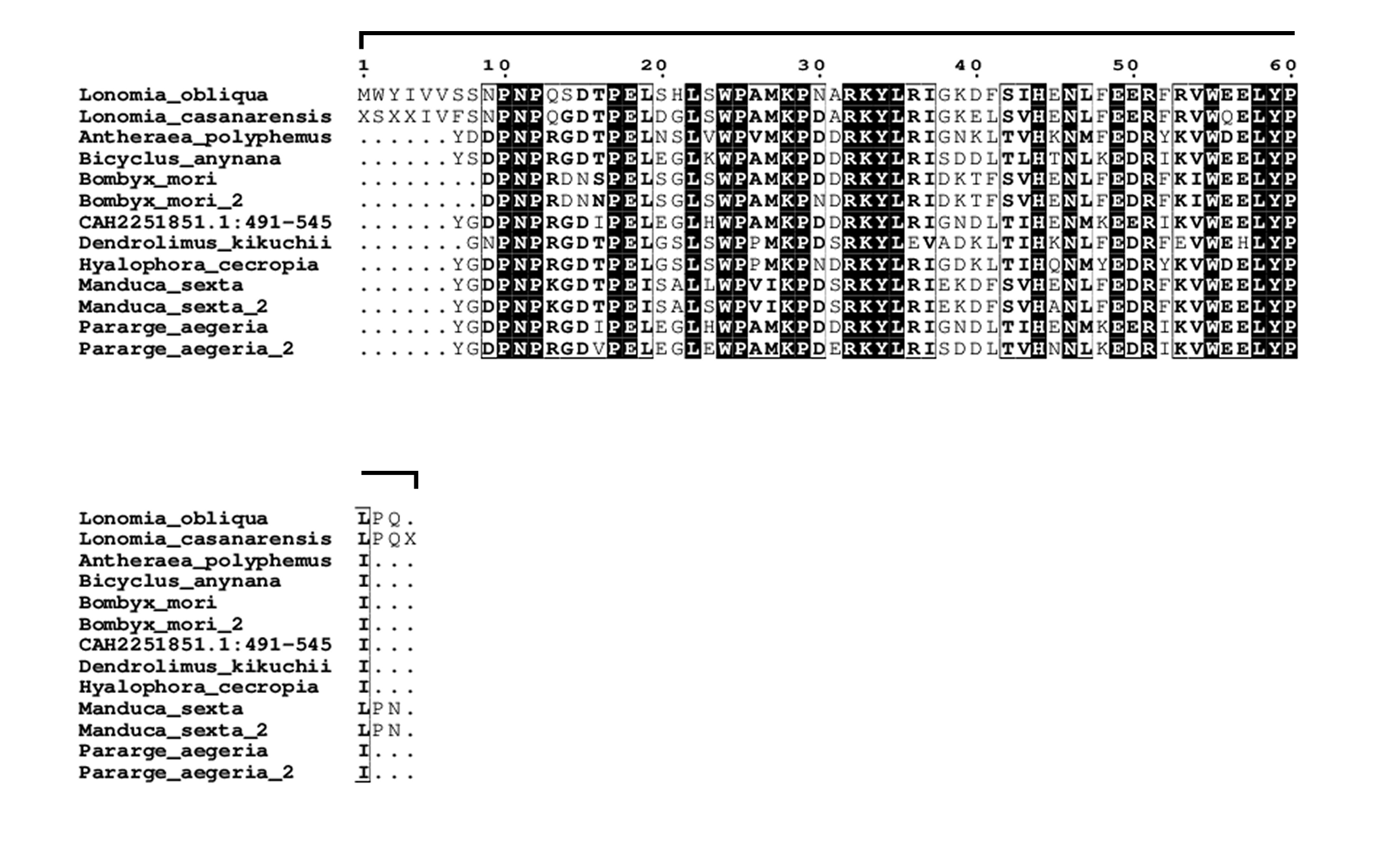

Supplement: jkag113_Supplementary_Data [file jkag113_supplementary_data.zip › FigS10_G3-2025-406412.png]

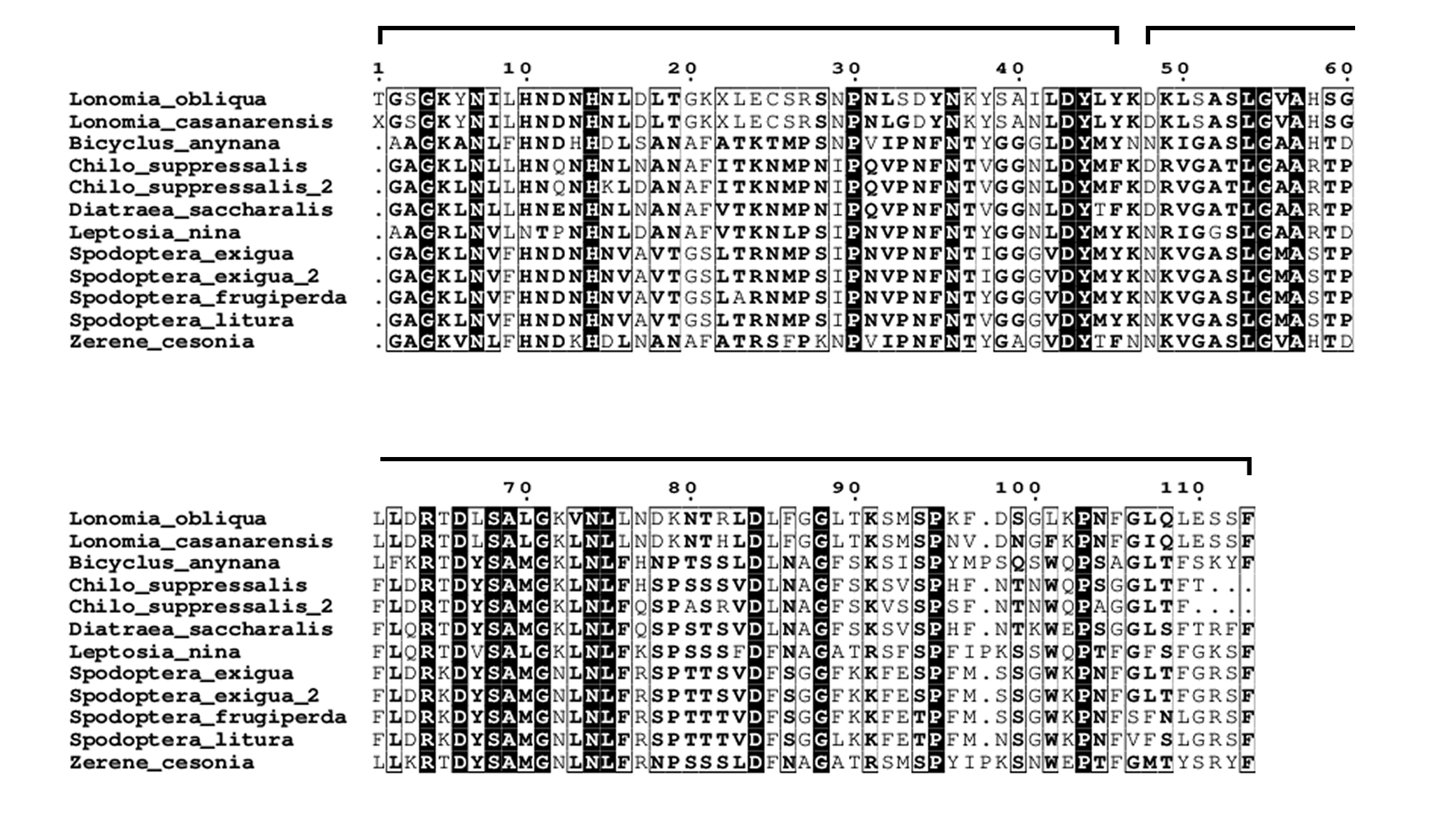

Supplement: jkag113_Supplementary_Data [file jkag113_supplementary_data.zip › FigS11_G3-2025-406412.png]

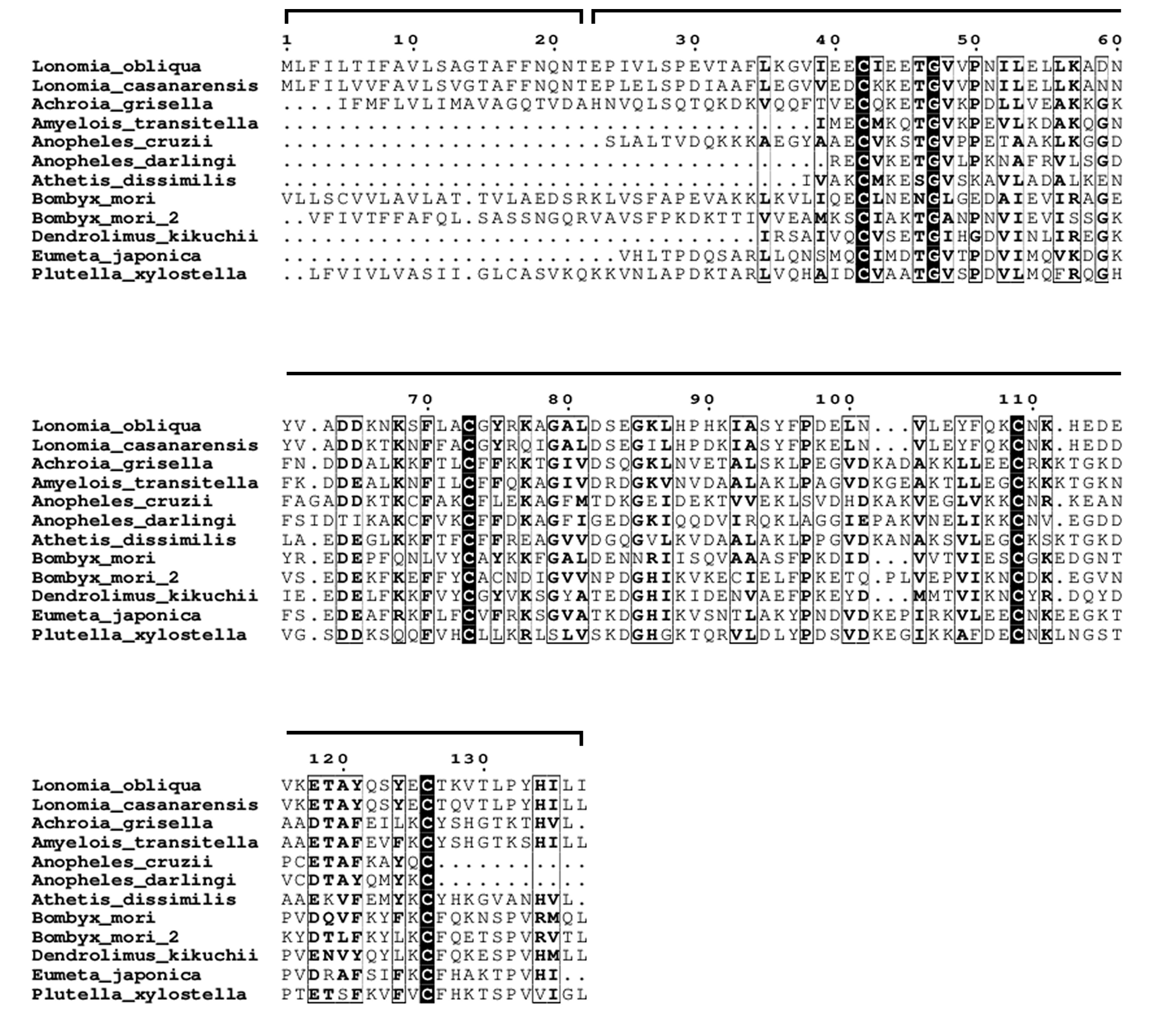

Supplement: jkag113_Supplementary_Data [file jkag113_supplementary_data.zip › FigS12_G3-2025-406412.png]

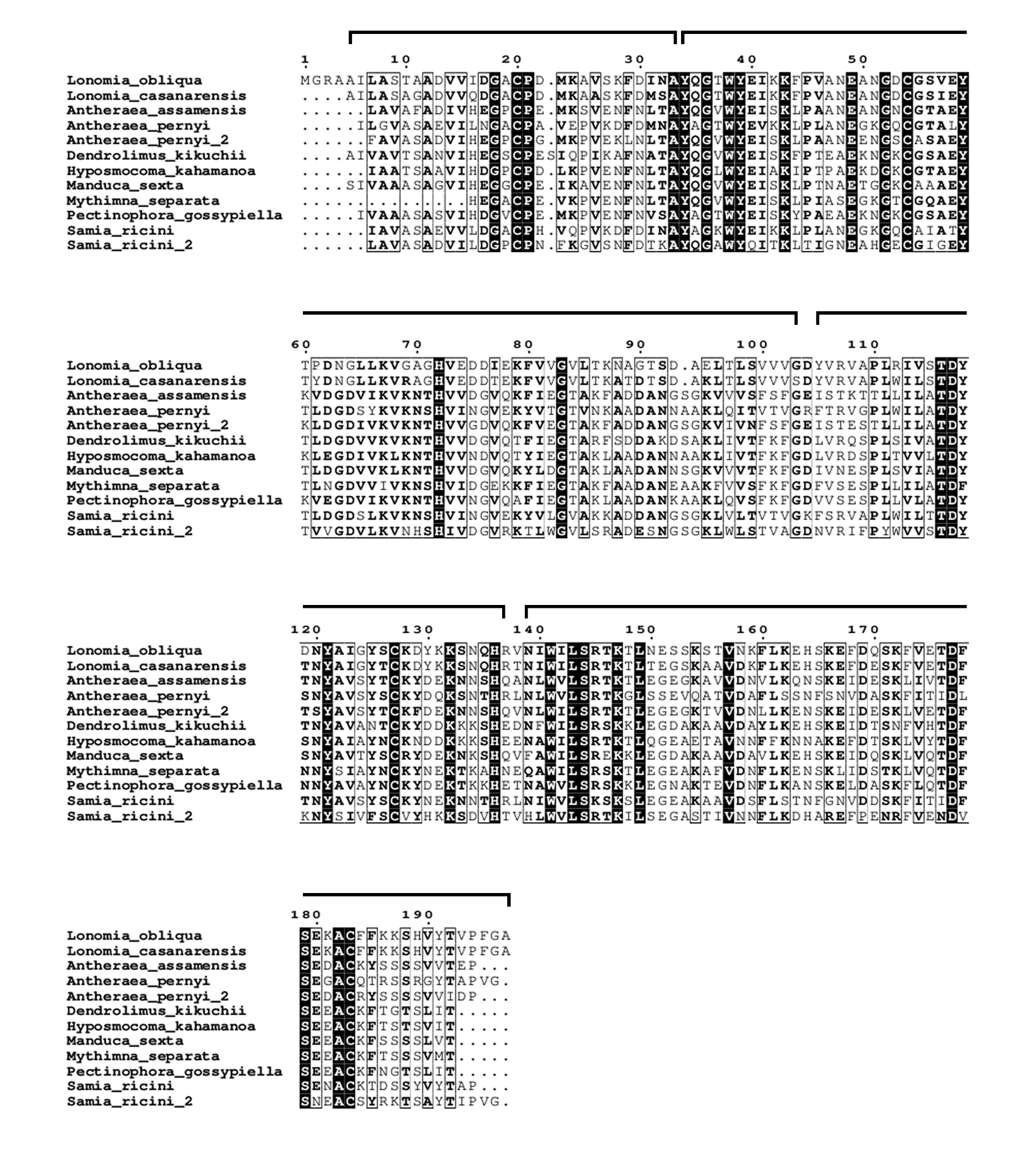

Supplement: jkag113_Supplementary_Data [file jkag113_supplementary_data.zip › FigS13_G3-2025-406412.png]

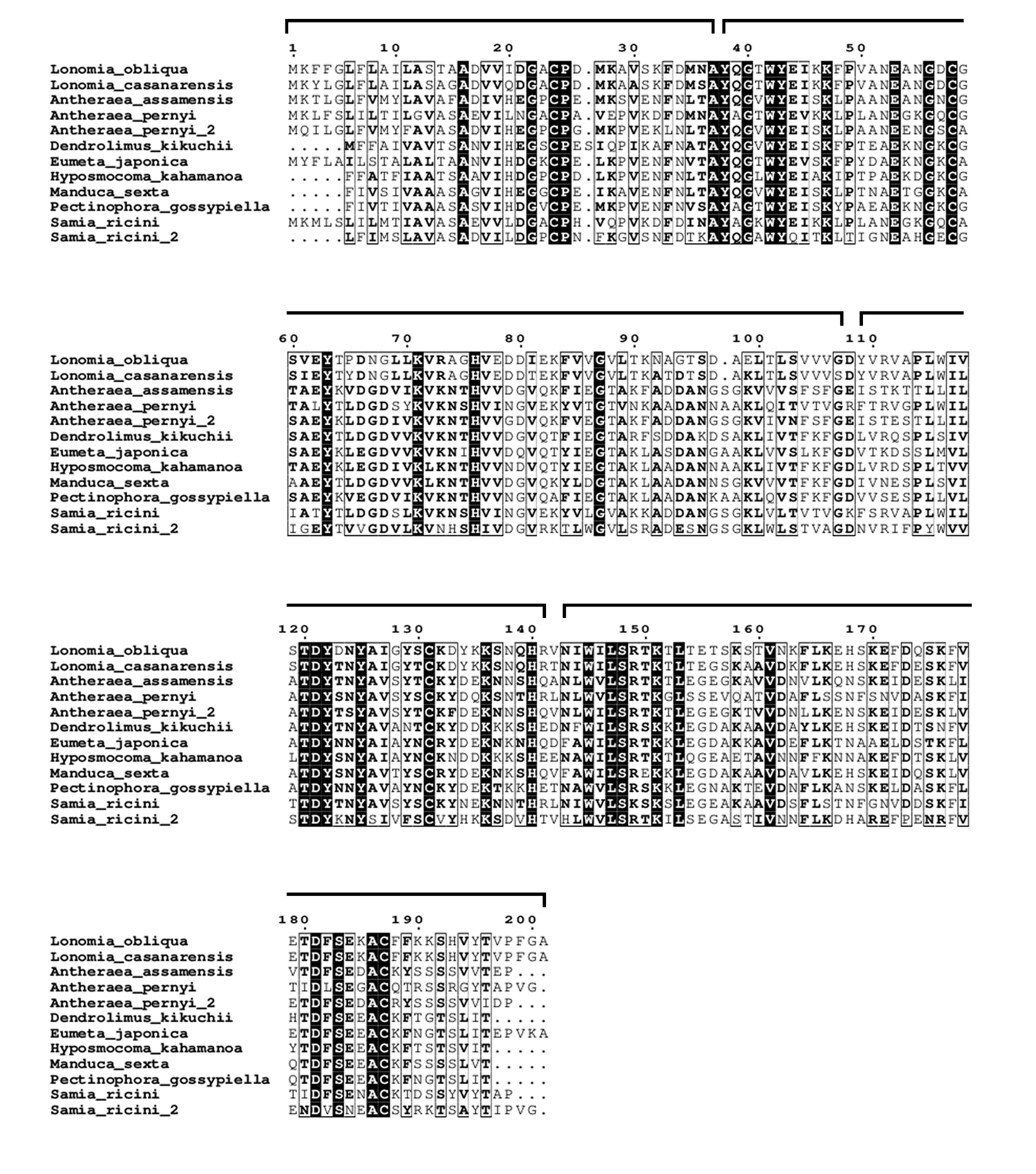

Supplement: jkag113_Supplementary_Data [file jkag113_supplementary_data.zip › FigS14_G3-2025-406412.png]

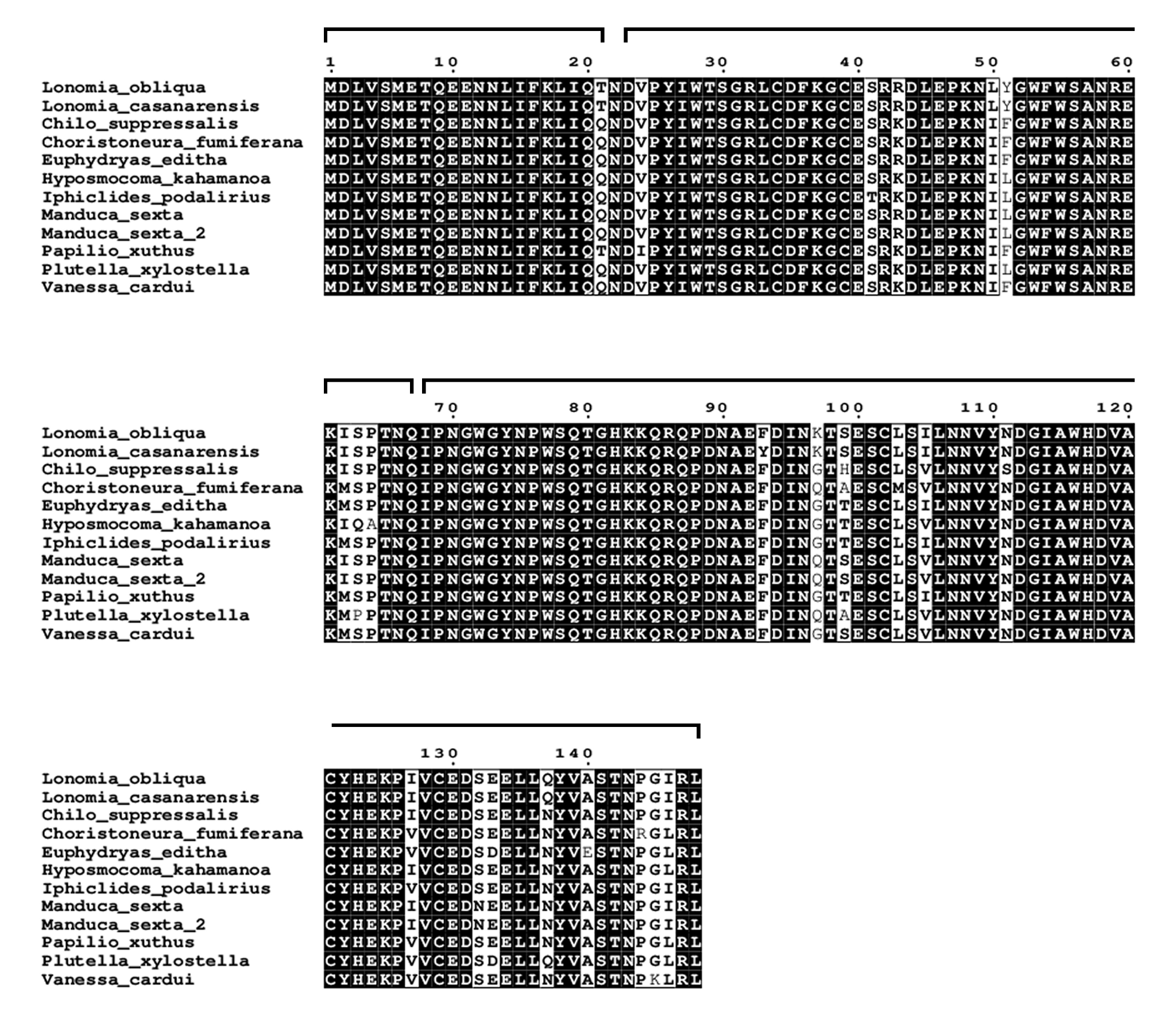

Supplement: jkag113_Supplementary_Data [file jkag113_supplementary_data.zip › FigS15_G3-2025-406412.png]

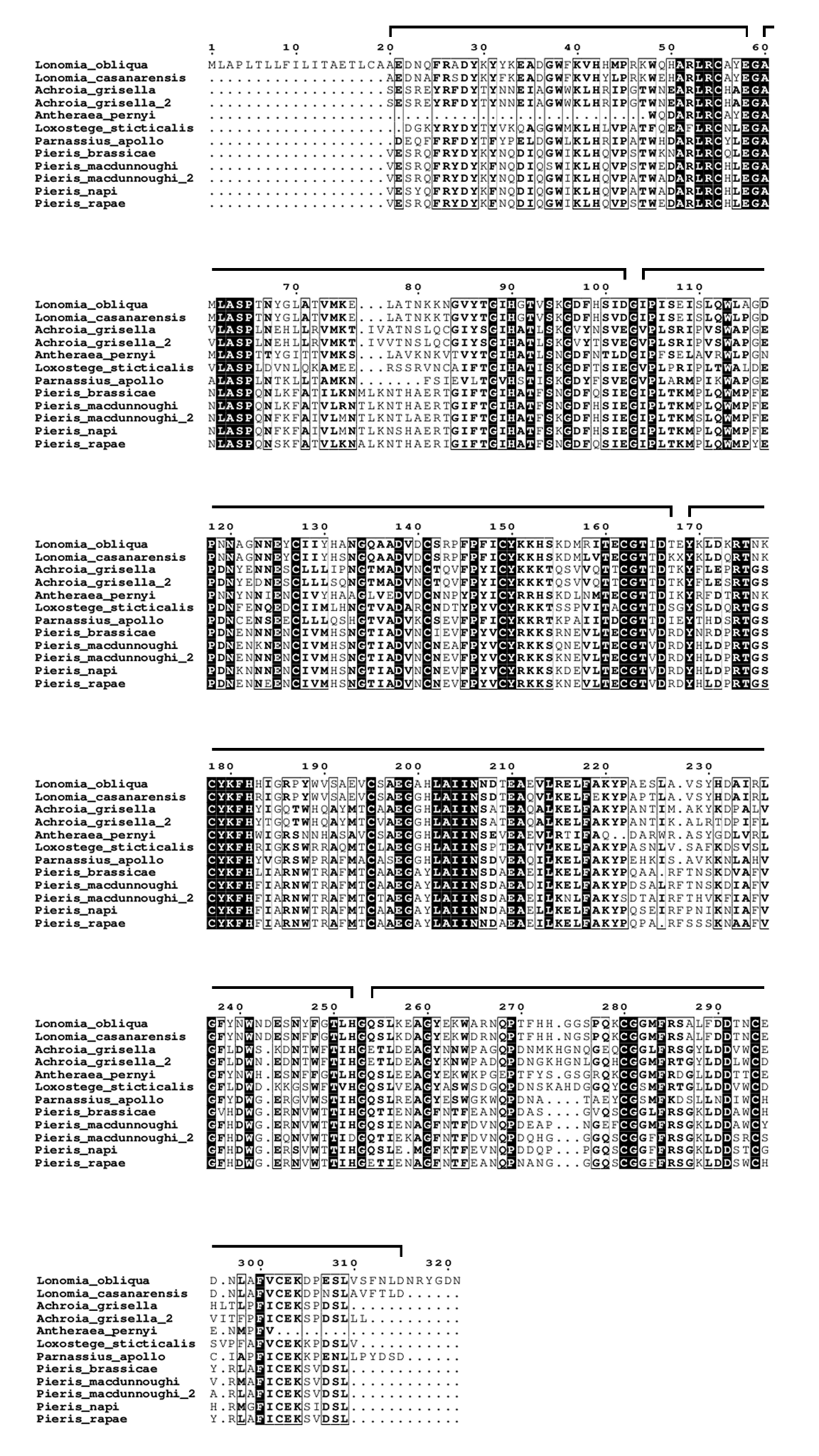

Supplement: jkag113_Supplementary_Data [file jkag113_supplementary_data.zip › FigS16_G3-2025-406412.png]

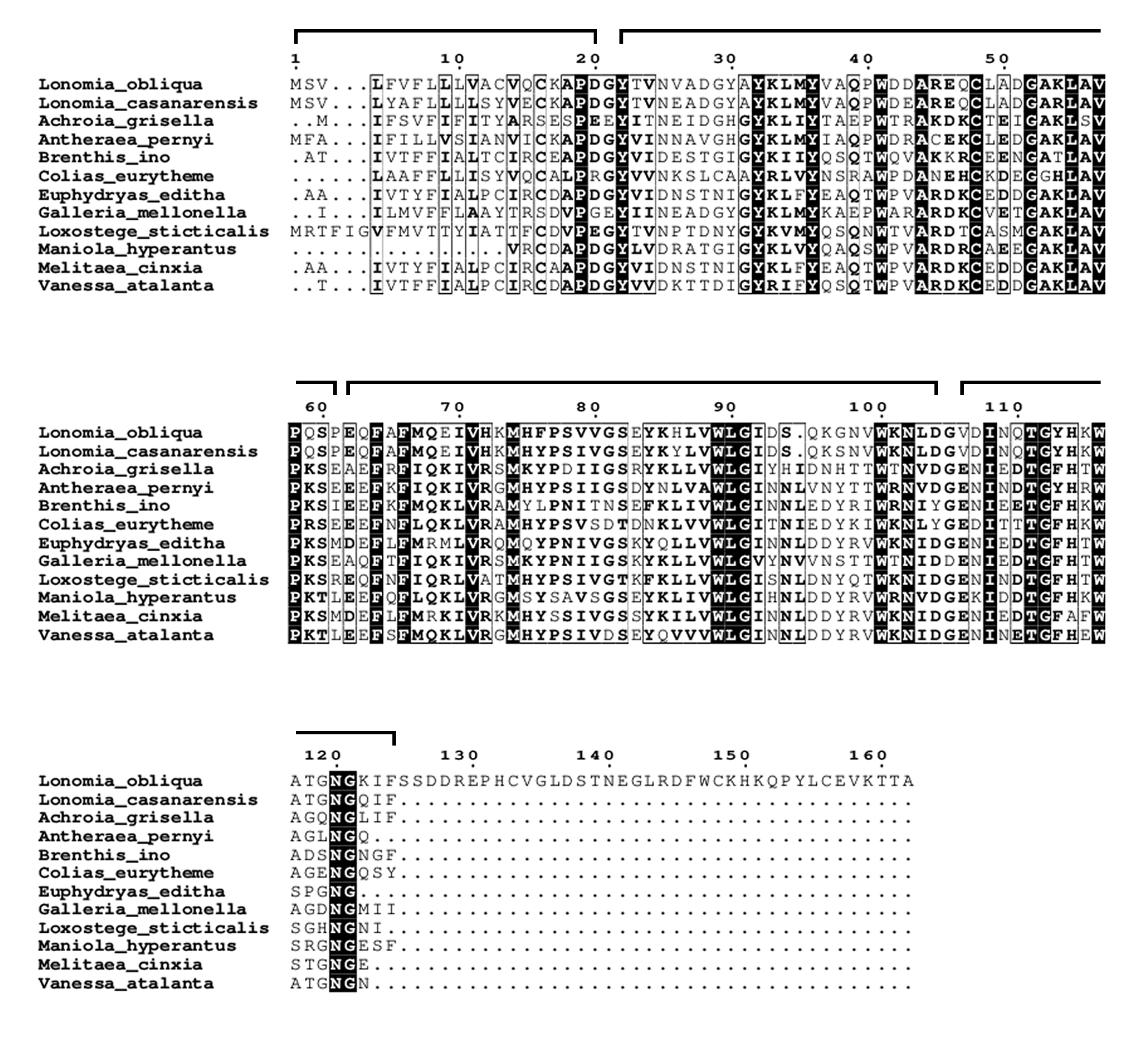

Supplement: jkag113_Supplementary_Data [file jkag113_supplementary_data.zip › FigS17_G3-2025-406412.png]

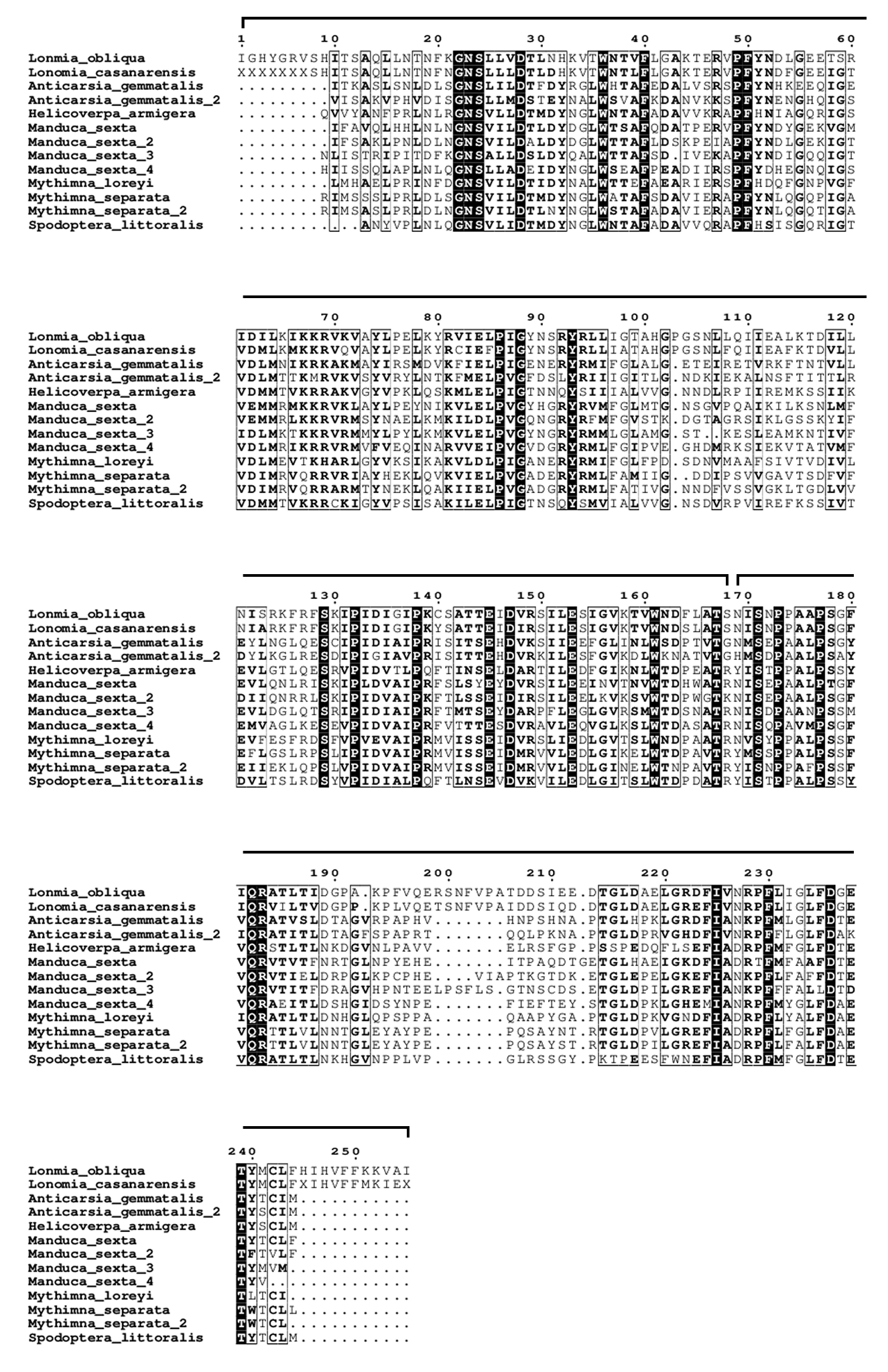

Supplement: jkag113_Supplementary_Data [file jkag113_supplementary_data.zip › FigS18_G3-2025-406412.png]

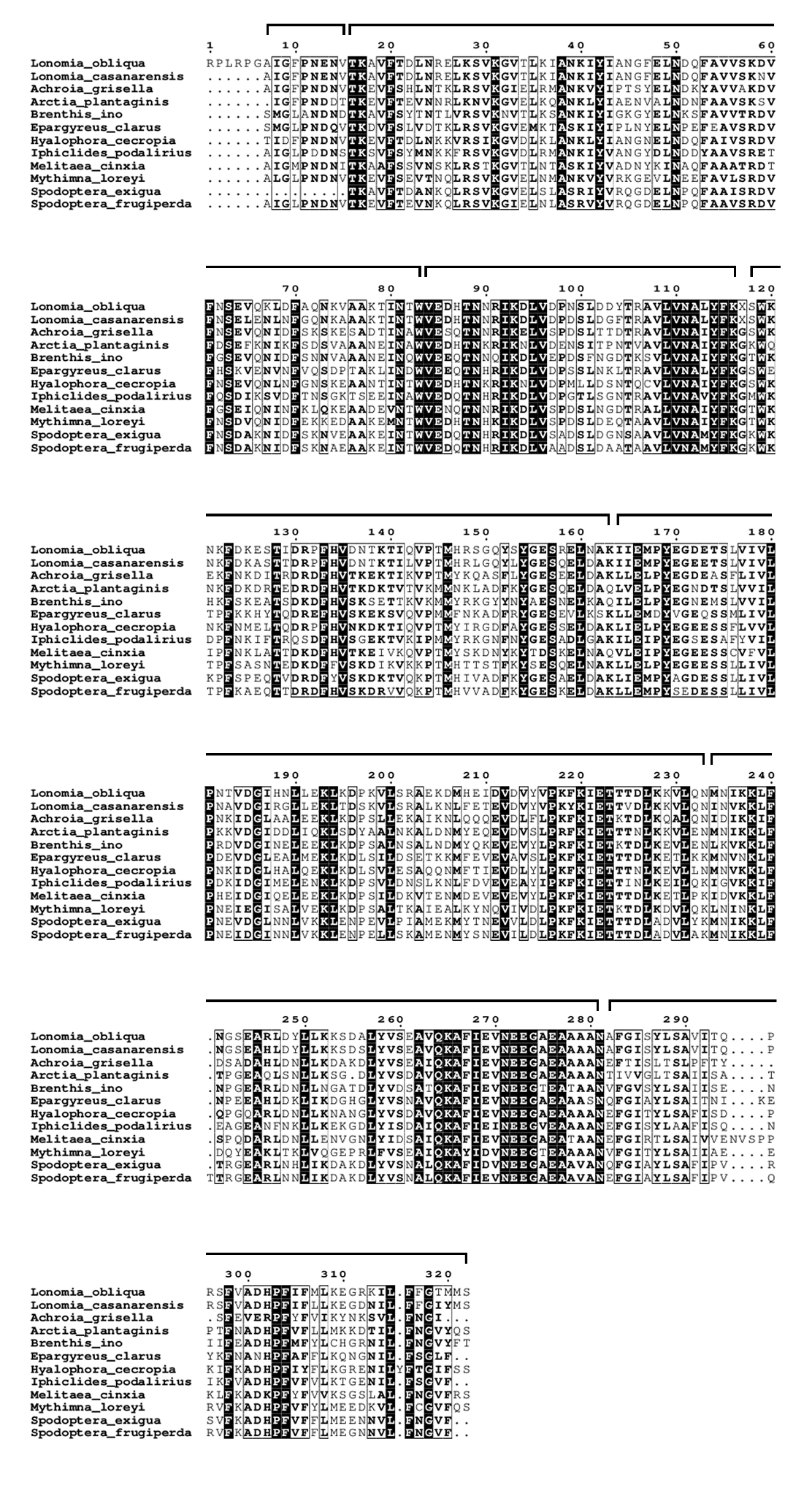

Supplement: jkag113_Supplementary_Data [file jkag113_supplementary_data.zip › FigS19_G3-2025-406412.png]

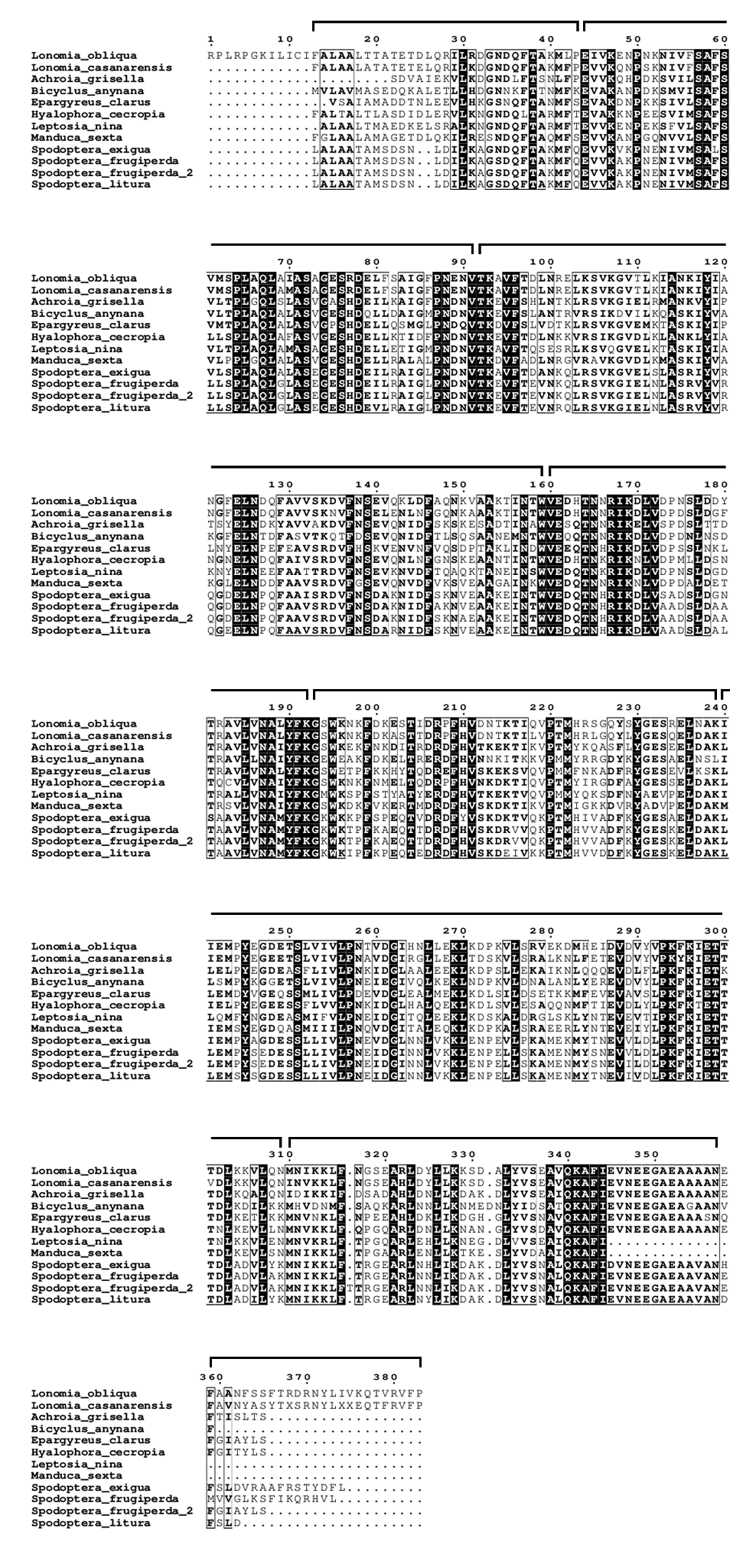

Supplement: jkag113_Supplementary_Data [file jkag113_supplementary_data.zip › FigS20_G3-2025-406412.png]

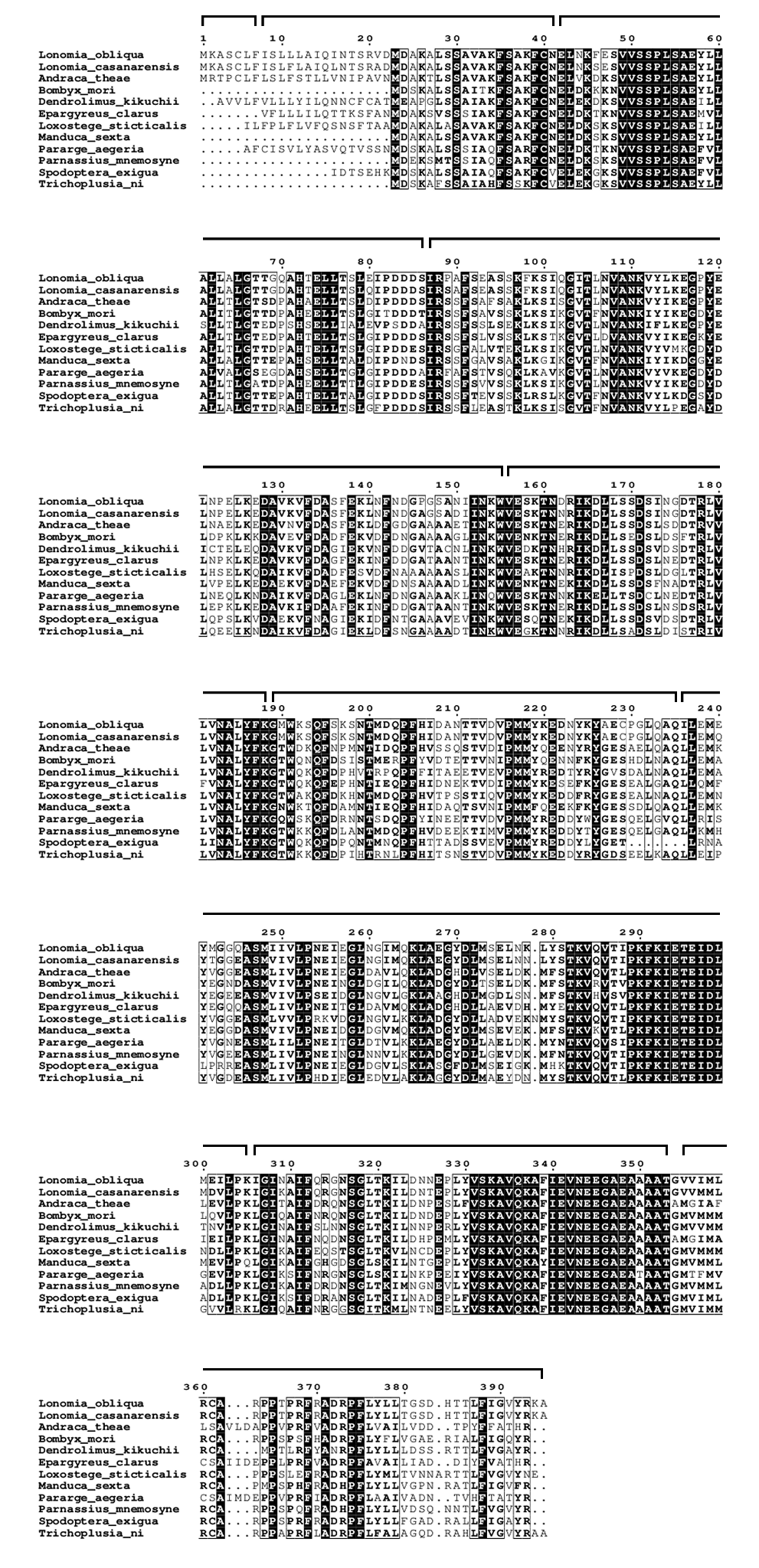

Supplement: jkag113_Supplementary_Data [file jkag113_supplementary_data.zip › FigS21_G3-2025-406412.png]

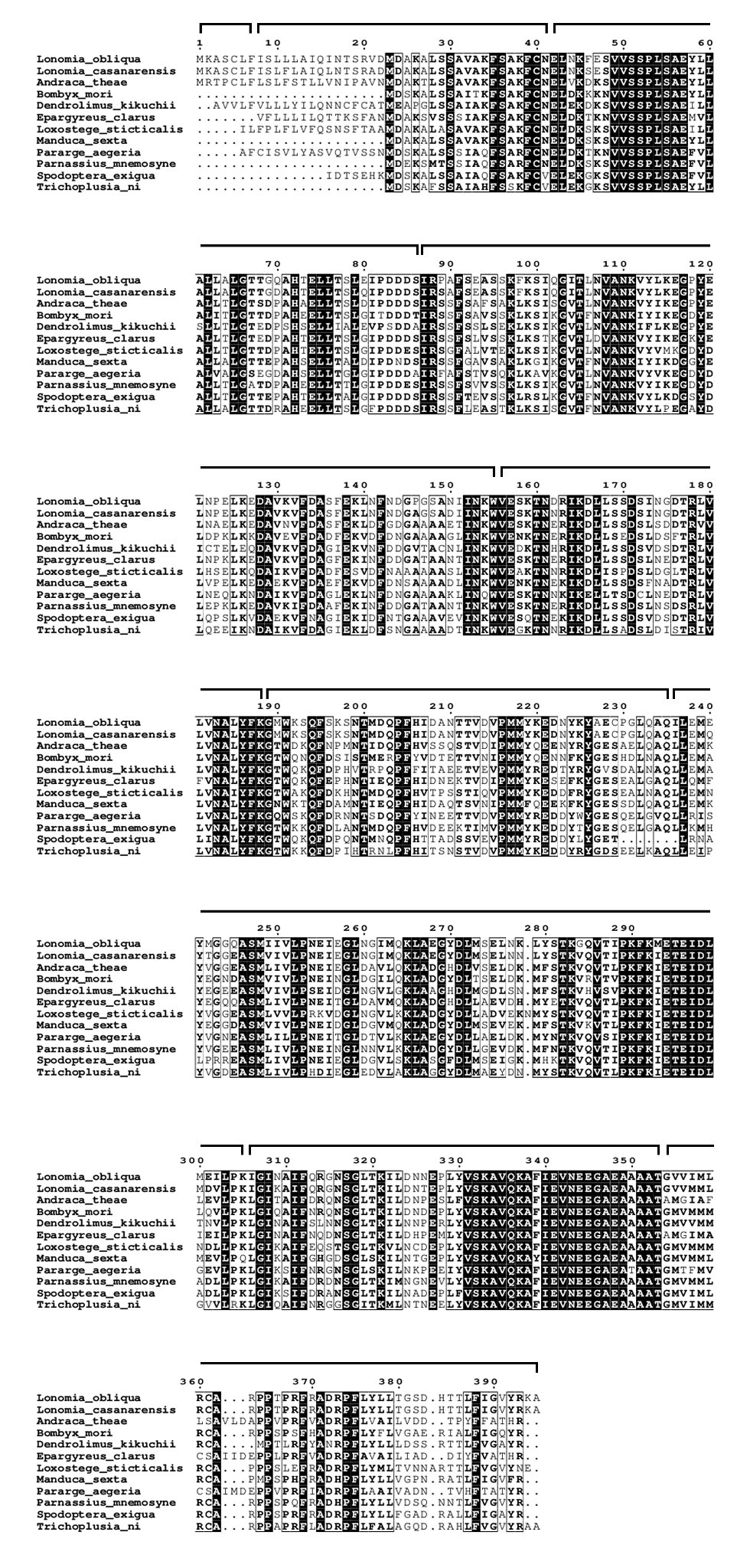

Supplement: jkag113_Supplementary_Data [file jkag113_supplementary_data.zip › FigS22_G3-2025-406412.png]

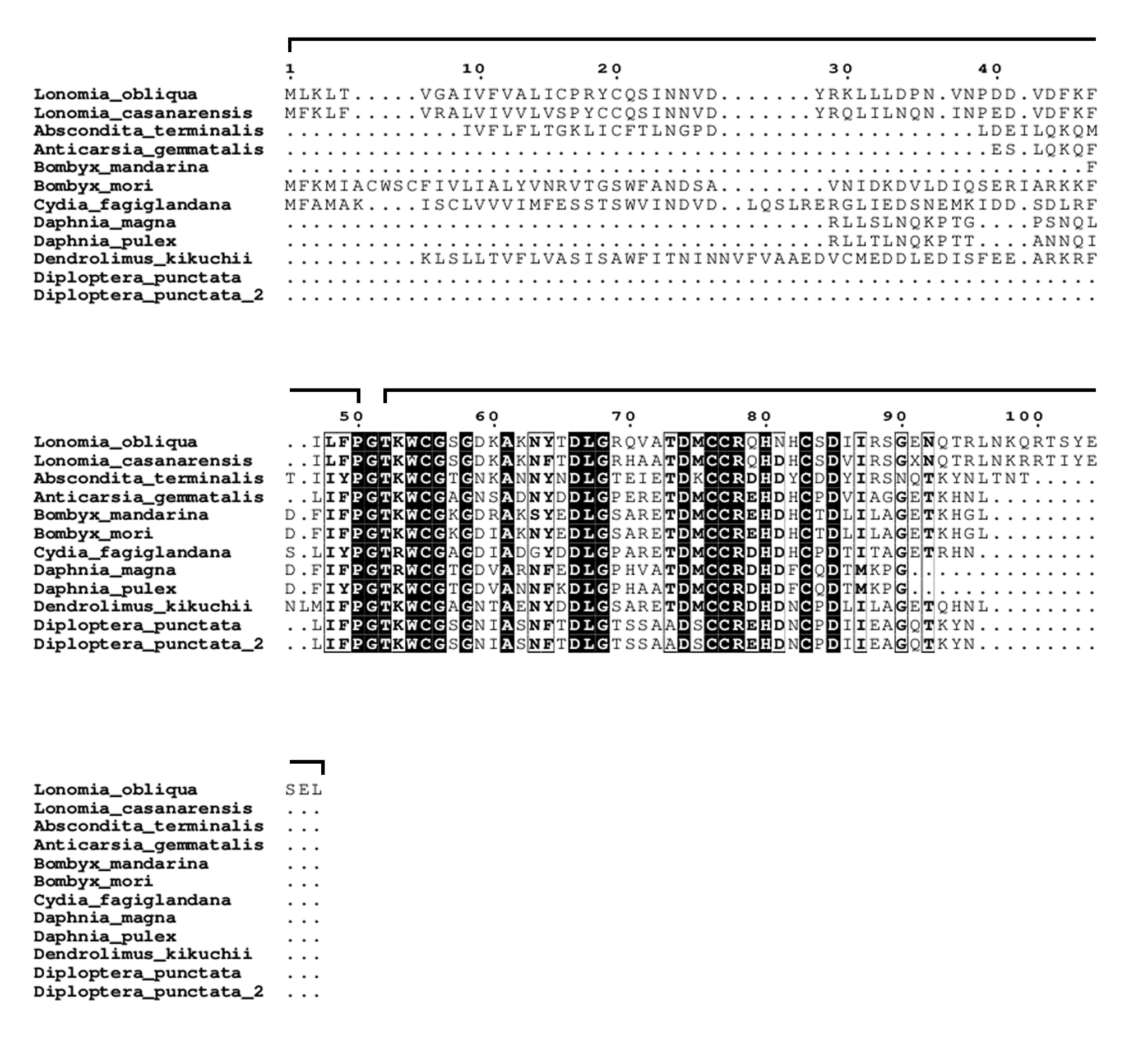

Supplement: jkag113_Supplementary_Data [file jkag113_supplementary_data.zip › FigS23_G3-2025-406412.png]

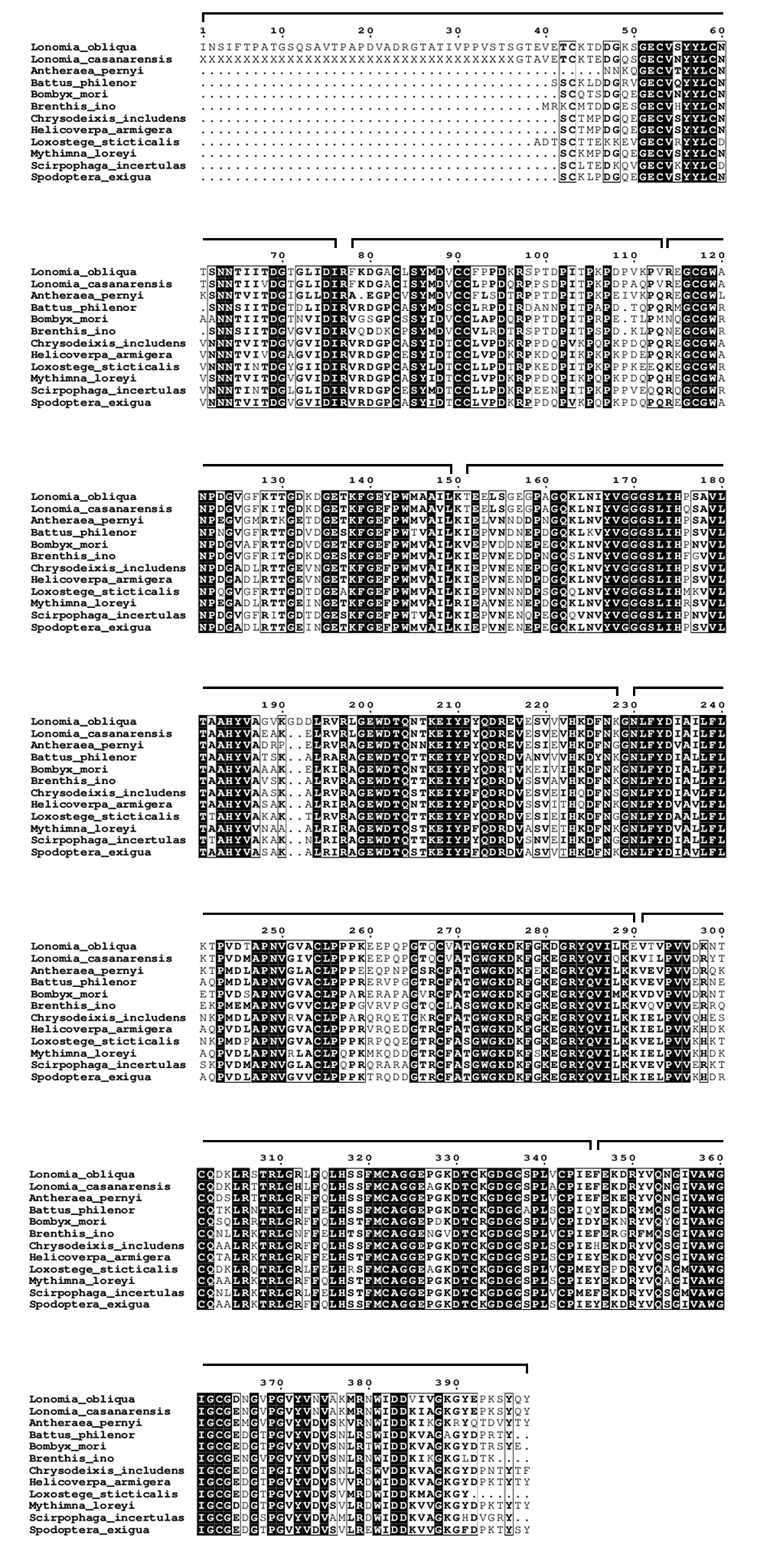

Supplement: jkag113_Supplementary_Data [file jkag113_supplementary_data.zip › FigS24_G3-2025-406412.png]

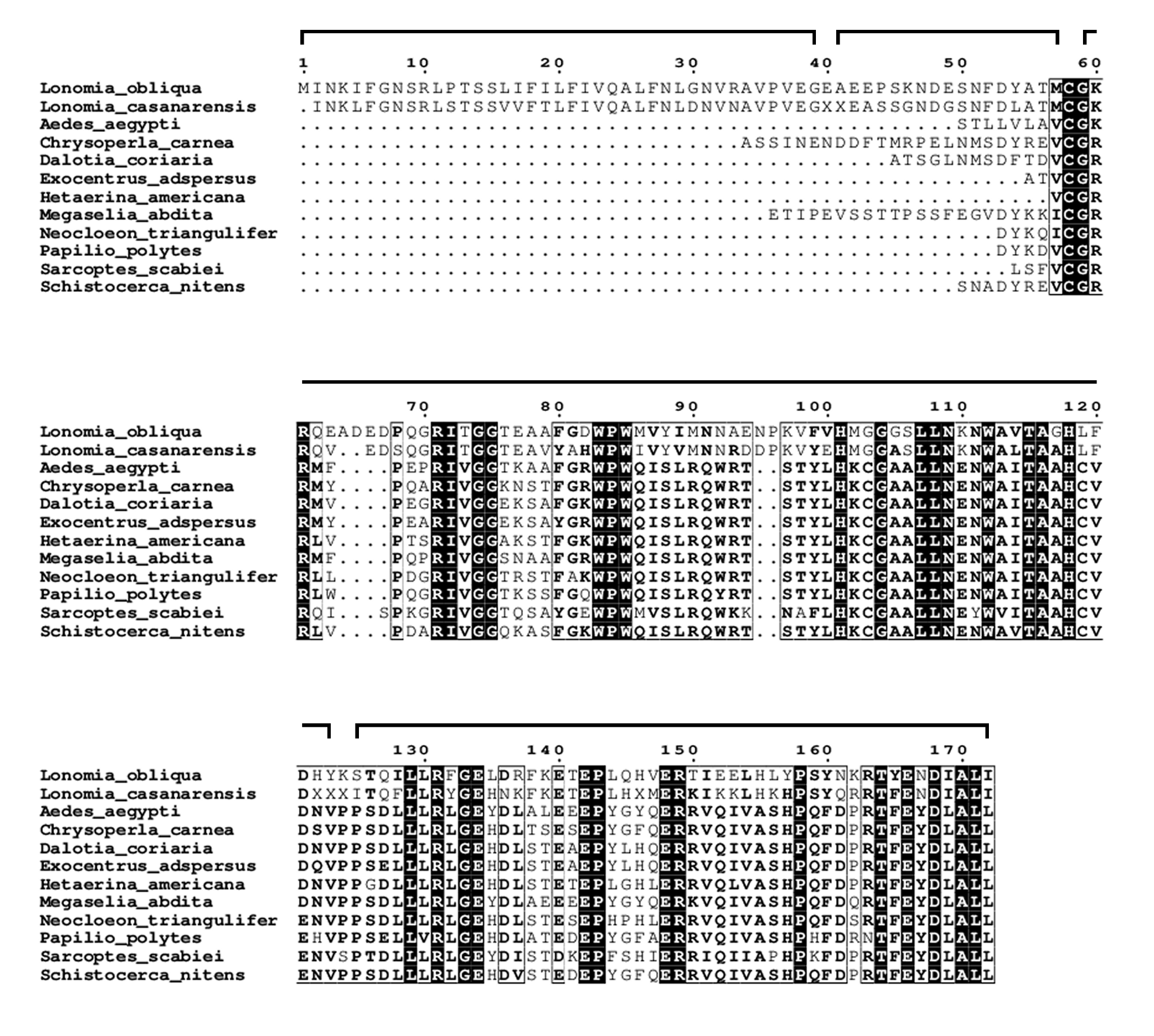

Supplement: jkag113_Supplementary_Data [file jkag113_supplementary_data.zip › FigS25_G3-2025-406412.png]

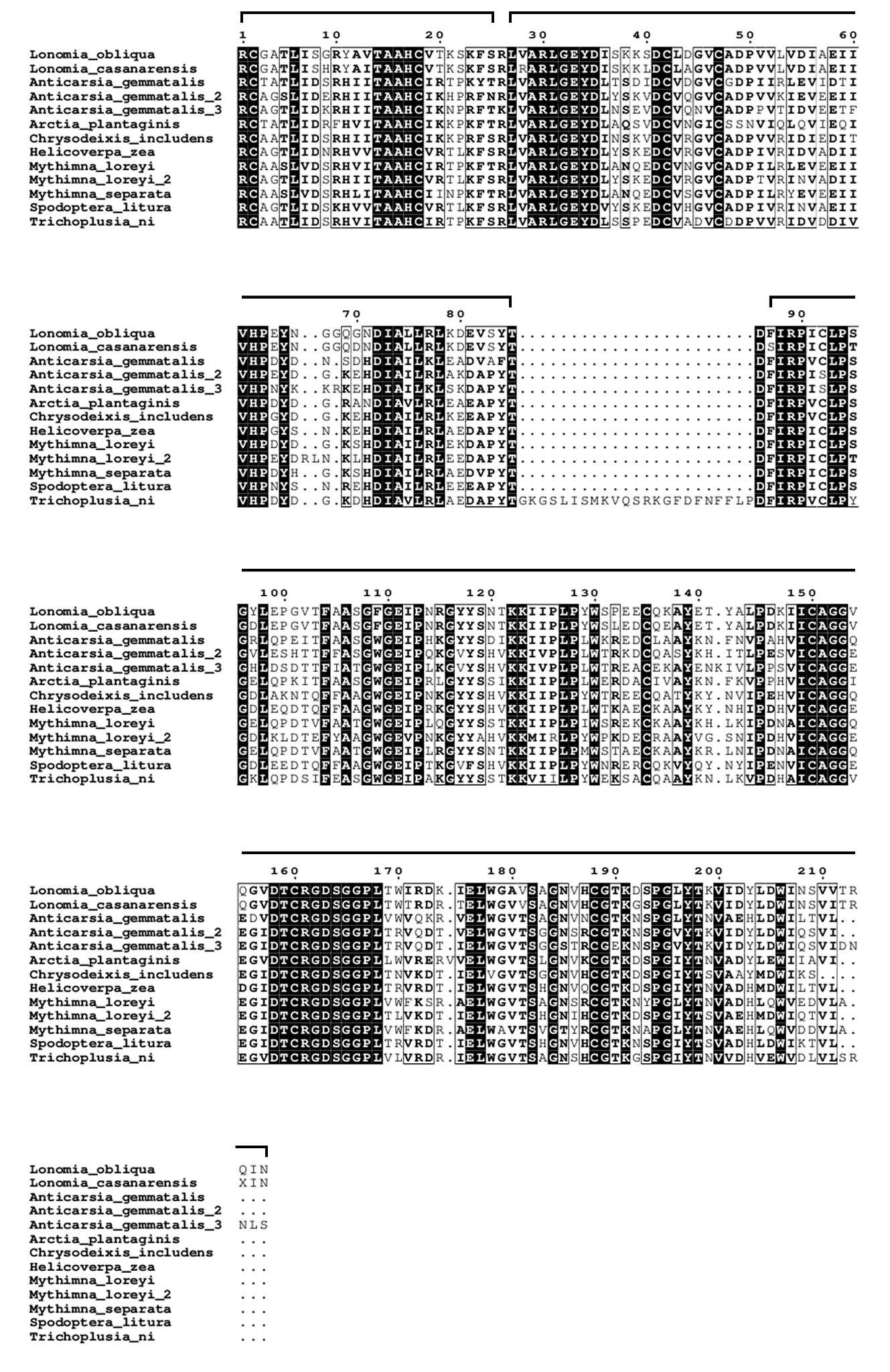

Supplement: jkag113_Supplementary_Data [file jkag113_supplementary_data.zip › FigS26_G3-2025-406412.png]

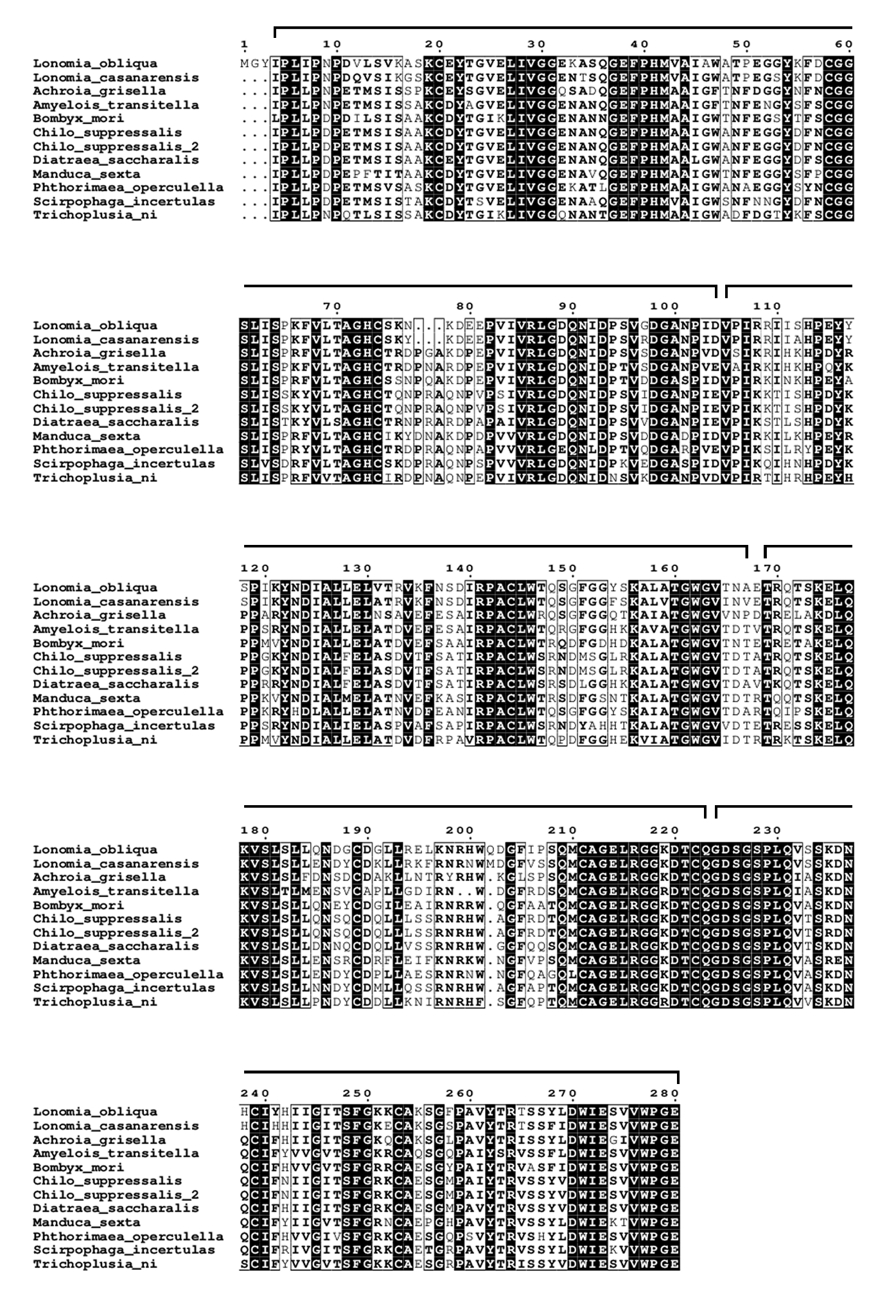

Supplement: jkag113_Supplementary_Data [file jkag113_supplementary_data.zip › FigS27_G3-2025-406412.png]

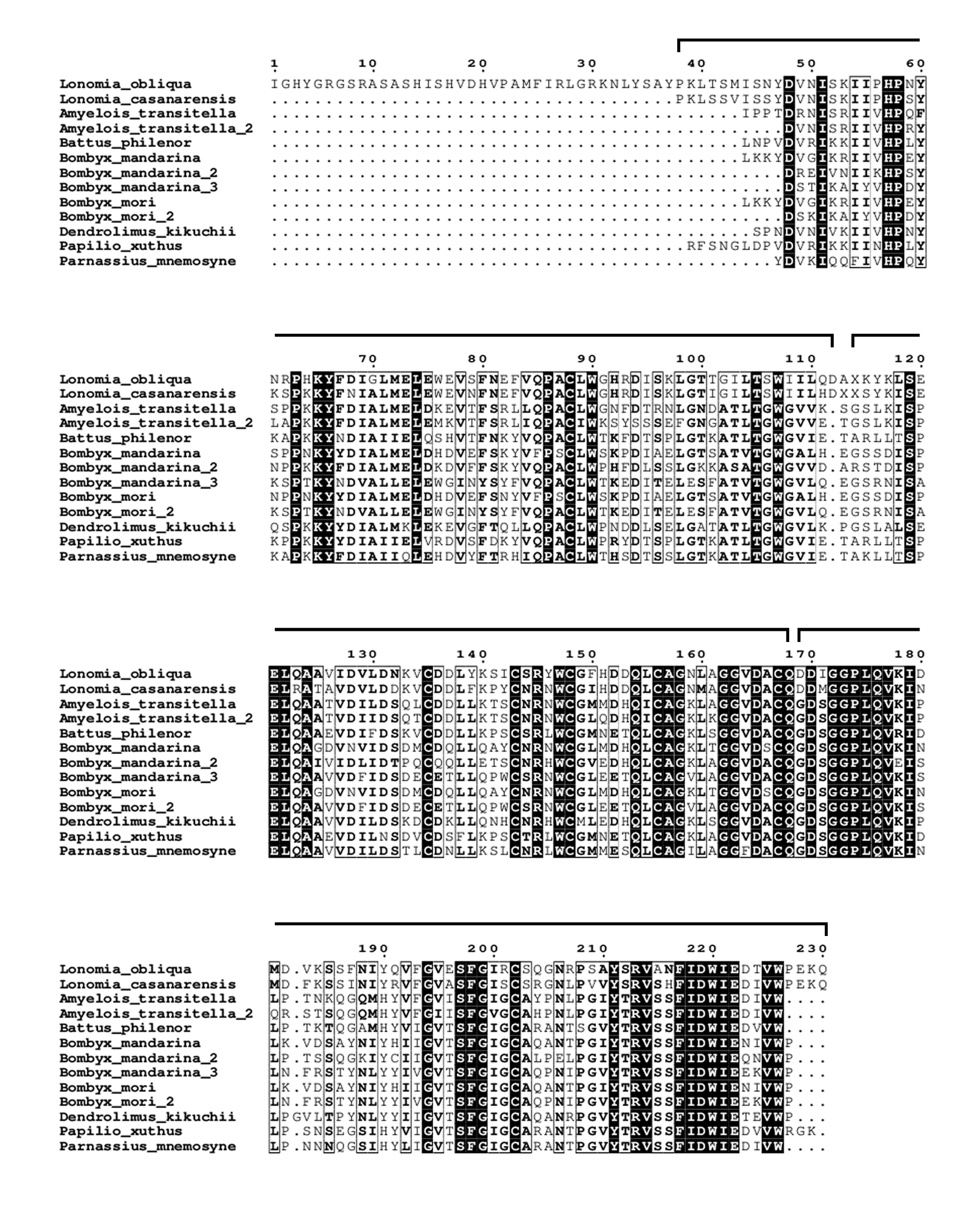

Supplement: jkag113_Supplementary_Data [file jkag113_supplementary_data.zip › FigS28_G3-2025-406412.png]

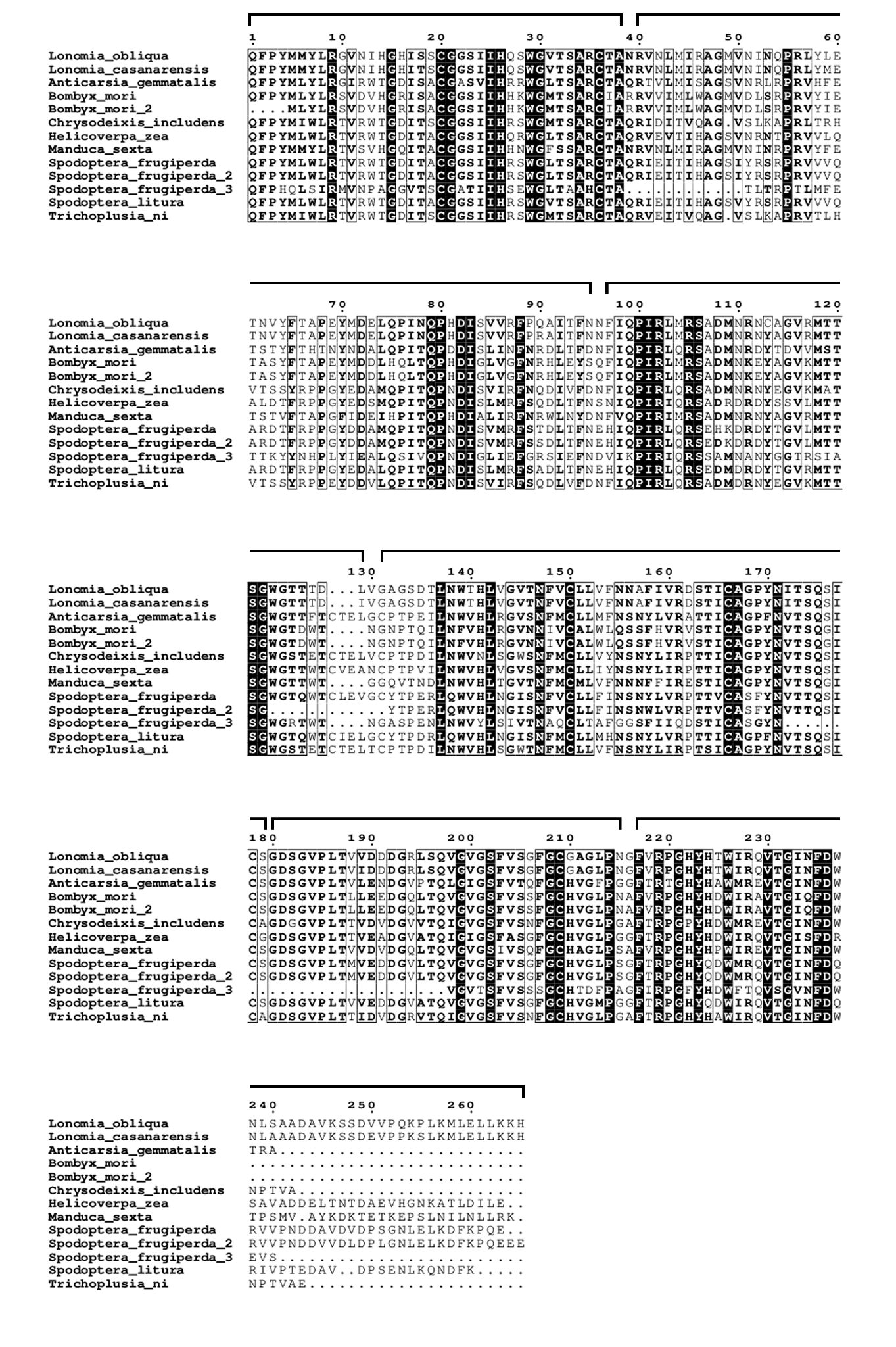

Supplement: jkag113_Supplementary_Data [file jkag113_supplementary_data.zip › FigS29_G3-2025-406412.png]

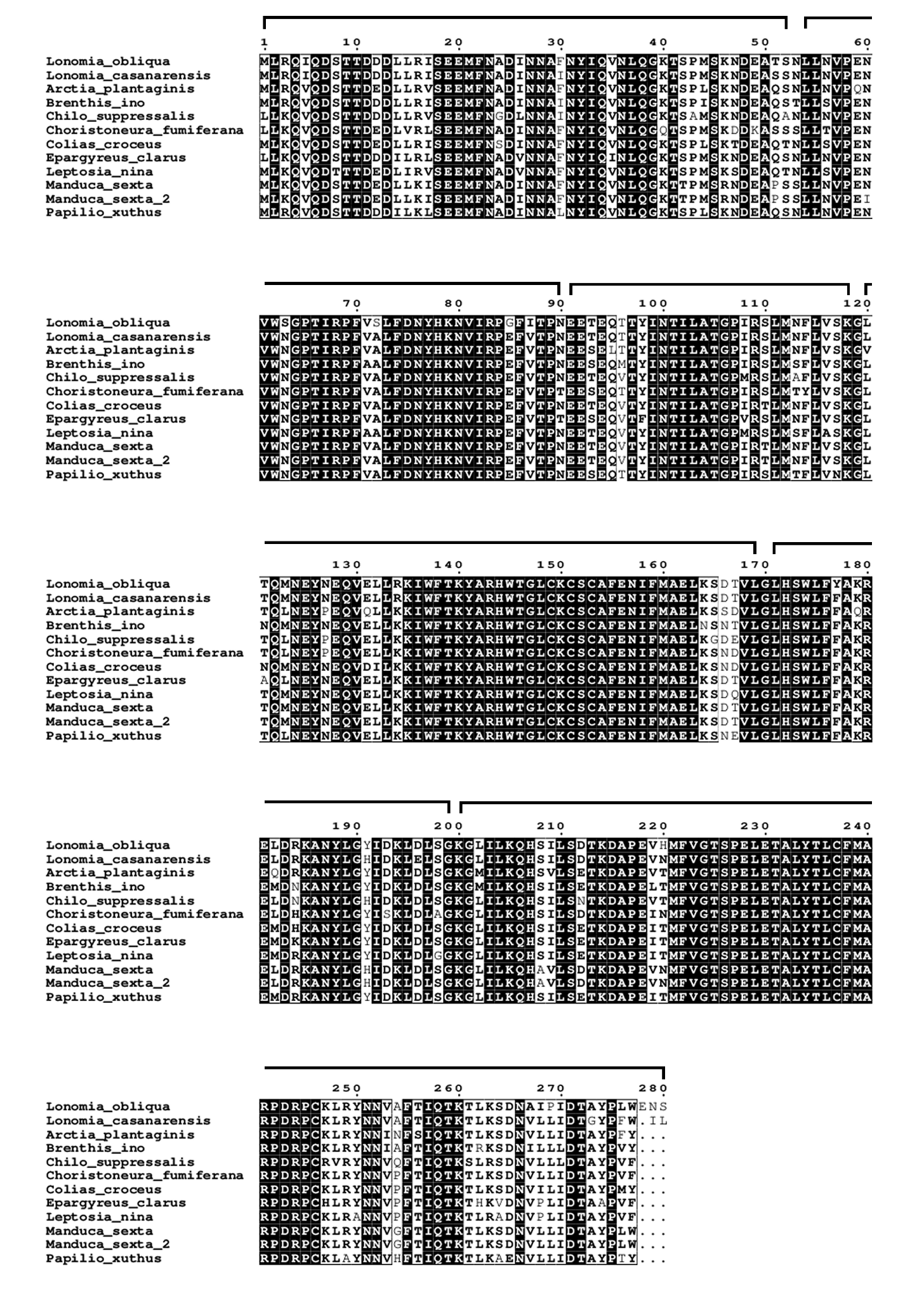

Supplement: jkag113_Supplementary_Data [file jkag113_supplementary_data.zip › FigS30_G3-2025-406412.png]

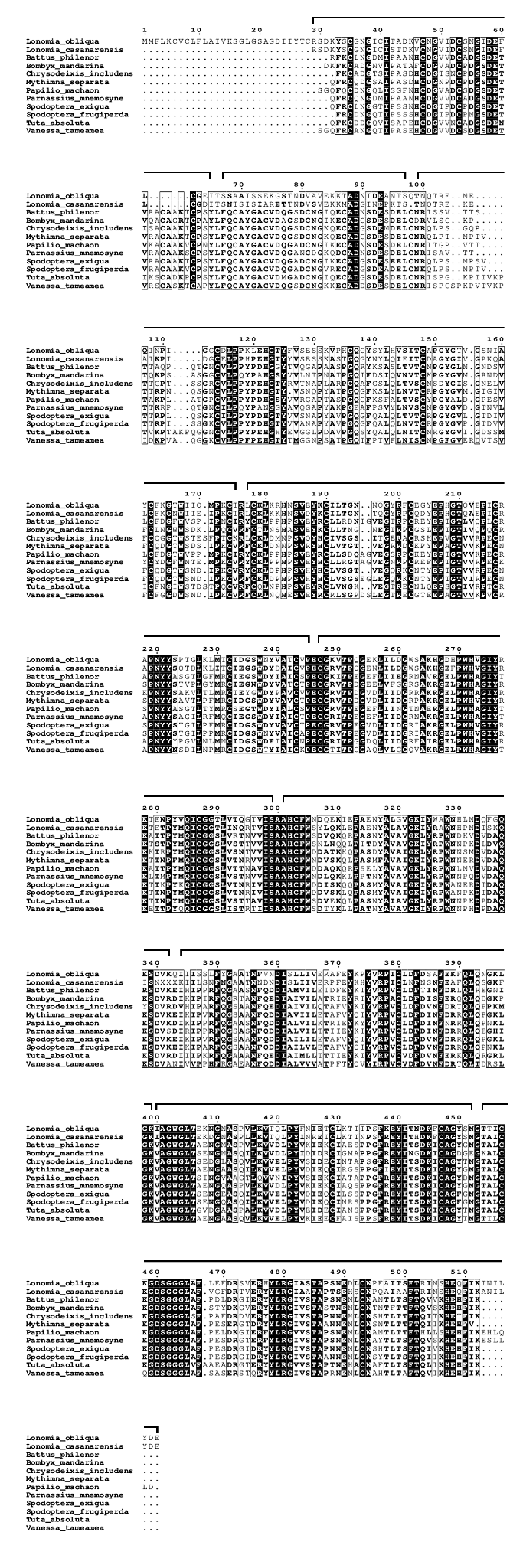

Supplement: jkag113_Supplementary_Data [file jkag113_supplementary_data.zip › FigS31_G3-2025-406412.png]

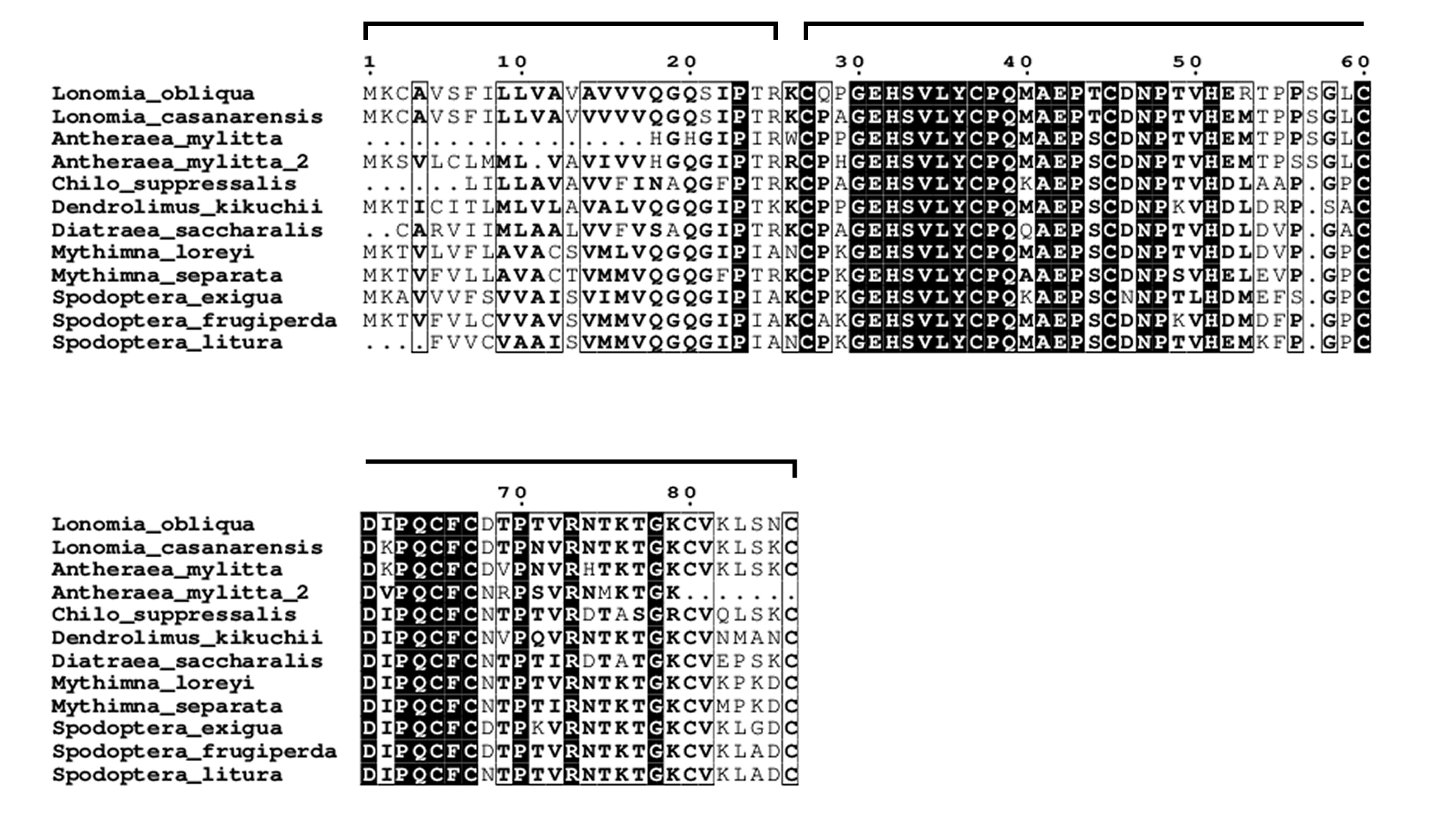

Supplement: jkag113_Supplementary_Data [file jkag113_supplementary_data.zip › FigS32_G3-2025-406412.png]

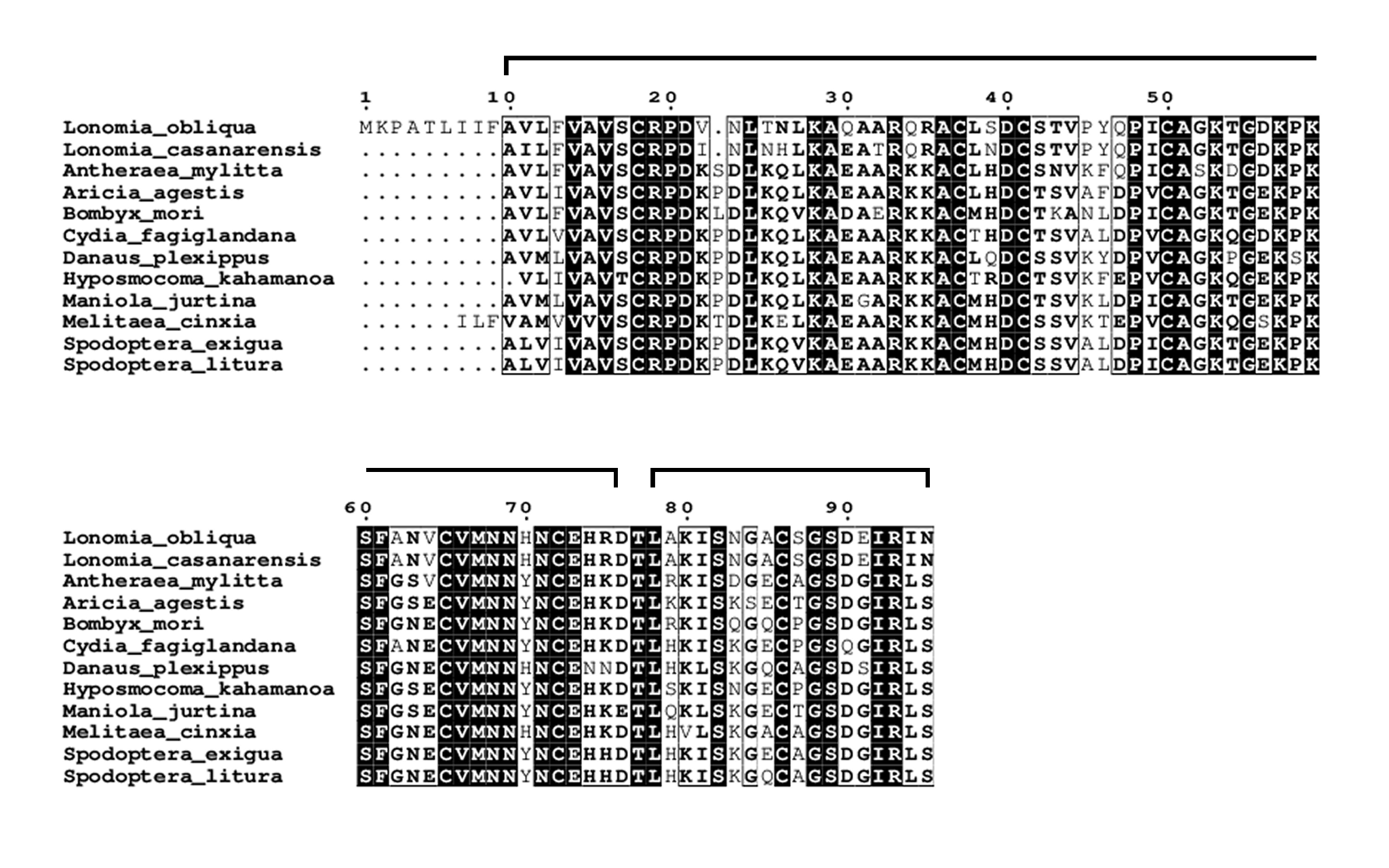

Supplement: jkag113_Supplementary_Data [file jkag113_supplementary_data.zip › FigS33_G3-2025-406412.png]

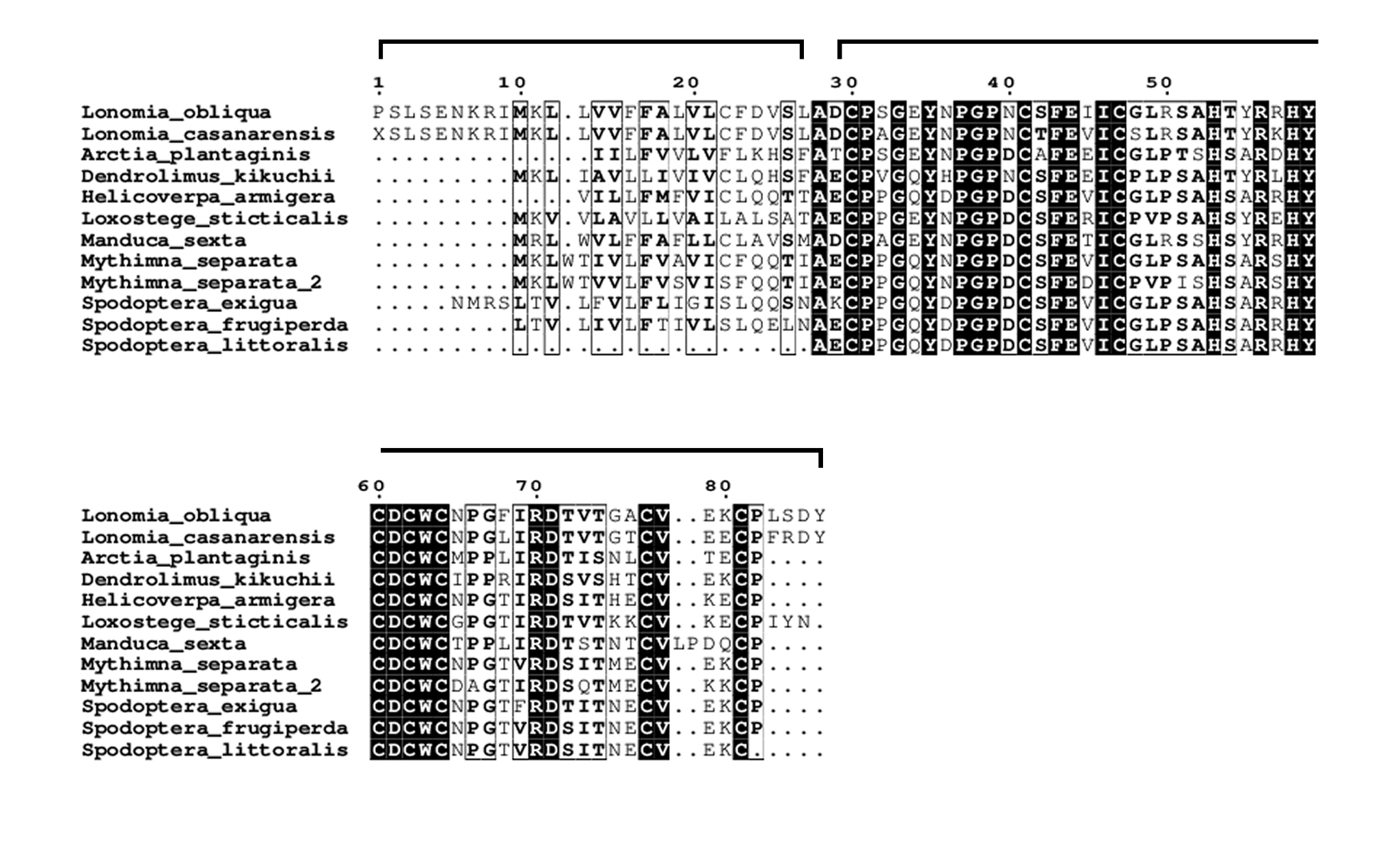

Supplement: jkag113_Supplementary_Data [file jkag113_supplementary_data.zip › FigS34_G3-2025-406412.png]

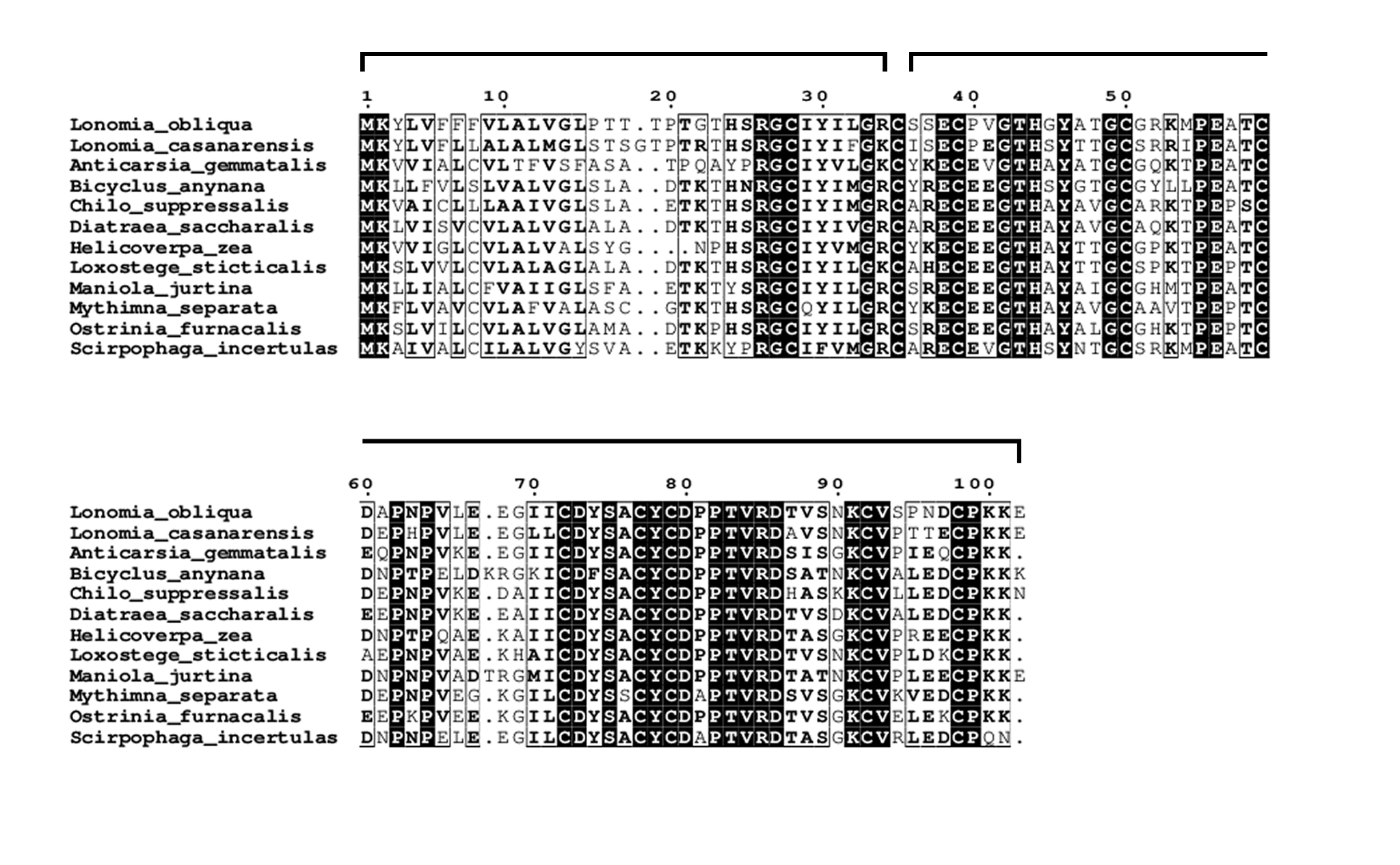

Supplement: jkag113_Supplementary_Data [file jkag113_supplementary_data.zip › FigS35_G3-2025-406412.png]

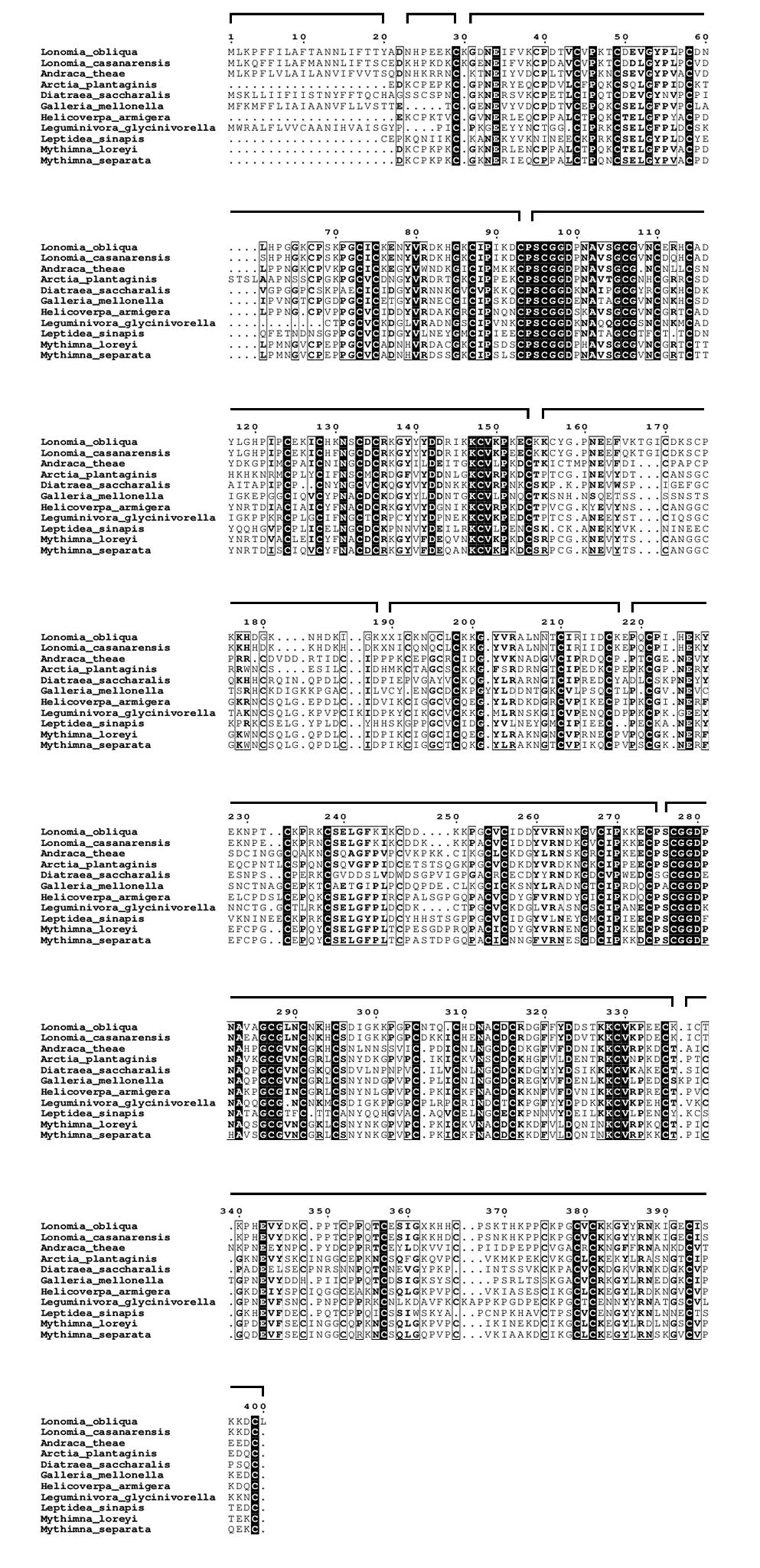

Supplement: jkag113_Supplementary_Data [file jkag113_supplementary_data.zip › FigS36_G3-2025-406412.png]
